# Electroconductive PEDOT nanoparticle integrated scaffolds for spinal cord tissue repair

**DOI:** 10.1186/s40824-022-00310-5

**Published:** 2022-11-22

**Authors:** Aleksandra Serafin, Mario Culebras Rubio, Marta Carsi, Pilar Ortiz-Serna, Maria J. Sanchis, Atul K. Garg, J. Miguel Oliveira, Jacob Koffler, Maurice N. Collins

**Affiliations:** 1 School of Engineering, Bernal Institute, University of Limerick, Limerick, Ireland; 2 Materials Science Institute (ICMUV), Universitat de València, c/ Catedrático José, Beltrán 2, 46980 Paterna, Valencia, Spain; 3 Instituto de Automática E Informática Industrial, Universitat Politècnica de Valencia, 46022 Valencia, Spain; 4 Departamento de Termodinámica Aplicada, Instituto Tecnológico de La Energía, Universitat Politècnica de València, Camí de Vera S/N, 46022 Valencia, Spain; 5 Manufacturing Technology and Innovation Global Supply Chain, Johnson & Johnson, Bridgewater, NJ 08807 USA; 6 3B’s Research Group, I3Bs—Research Institute on Biomaterials, Biodegradables and Biomimetics, University of Minho, Headquarters of the European Institute of Excellence On Tissue Engineering and Regenerative Medicine, AveParkParque de Ciência E Tecnologia, Zona Industrial da Gandra, Barco, 4805-017 Guimarães, Portugal; 7 ICVS/3B’s—PT Government Associate Laboratory, Braga, Braga 4710-057 Portugal; 8 Department of Neuroscience, University of California San Diego, La Jolla, CA 92093 USA; 9 Veterans Affairs Medical Center, San Diego, CA USA; 10 Health Research Institute and AMBER, University of Limerick, Limerick, Ireland

**Keywords:** Electroconductive scaffolds, PEDOT nanoparticles, Tissue engineering, Spinal cord injury

## Abstract

**Background:**

Hostile environment around the lesion site following spinal cord injury (SCI) prevents the re-establishment of neuronal tracks, thus significantly limiting the regenerative capability. Electroconductive scaffolds are emerging as a promising option for SCI repair, though currently available conductive polymers such as polymer poly(3,4-ethylenedioxythiophene) polystyrene sulfonate (PEDOT:PSS) present poor biofunctionality and biocompatibility, thus limiting their effective use in SCI tissue engineering (TE) treatment strategies.

**Methods:**

PEDOT NPs were synthesized via chemical oxidation polymerization in miniemulsion. The conductive PEDOT NPs were incorporated with gelatin and hyaluronic acid (HA) to create gel:HA:PEDOT-NPs scaffolds. Morphological analysis of both PEDOT NPs and scaffolds was conducted via SEM. Further characterisation included dielectric constant and permittivity variances mapped against morphological changes after crosslinking, Young’s modulus, FTIR, DLS, swelling studies, rheology, in-vitro, and in-vivo biocompatibility studies were also conducted.

**Results:**

Incorporation of PEDOT NPs increased the conductivity of scaffolds to 8.3 × 10^–4^ ± 8.1 × 10^–5^ S/cm. The compressive modulus of the scaffold was tailored to match the native spinal cord at 1.2 ± 0.2 MPa, along with controlled porosity. Rheological studies of the hydrogel showed excellent 3D shear-thinning printing capabilities and shape fidelity post-printing. In-vitro studies showed the scaffolds are cytocompatible and an in-vivo assessment in a rat SCI lesion model shows glial fibrillary acidic protein (GFAP) upregulation not directly in contact with the lesion/implantation site, with diminished astrocyte reactivity. Decreased levels of macrophage and microglia reactivity at the implant site is also observed. This positively influences the re-establishment of signals and initiation of healing mechanisms. Observation of axon migration towards the scaffold can be attributed to immunomodulatory properties of HA in the scaffold caused by a controlled inflammatory response. HA limits astrocyte activation through its CD44 receptors and therefore limits scar formation. This allows for a superior axonal migration and growth towards the targeted implantation site through the provision of a stimulating microenvironment for regeneration.

**Conclusions:**

Based on these results, the incorporation of PEDOT NPs into Gel:HA biomaterial scaffolds enhances not only the conductive capabilities of the material, but also the provision of a healing environment around lesions in SCI. Hence, gel:HA:PEDOT-NPs scaffolds are a promising TE option for stimulating regeneration for SCI.

**Supplementary Information:**

The online version contains supplementary material available at 10.1186/s40824-022-00310-5.

## Background

Spinal cord injury (SCI) is a traumatic event which often results in the loss of sensory and motor function, at or below the site of injury. About 12,000 new cases of SCI are reported every year in the United States alone, but due to the present clinical inability to successfully treat SCI, the amount of people suffering from this ailment is ever increasing [1].

Initial SCI is followed by a cascade of events, including tissue necrosis, haemorrhage, oedema, and lesion formation around the injury site [2]. Upregulation of reactive astrocytes by means of upregulated expression of glial fibrillary acidic protein (GFAP) is one of the primary inflammatory factors. Microglia, macrophages and oligodendrocyte precursor cells aid in the formation of glial scarring around the lesion site [3]. Still, there is much scientific debate on the influence of the glial scar on SCI regeneration [4, 5]. The inability of the central nervous system (CNS) cells to regenerate functional capabilities across the lesion site due to the hostile environment following injury is still a problem of major importance in SCI repair [4, 5].

Tissue engineered (TE) scaffolds offer a potential solution by aiming to bridge the neuronal gap created as result of SCI. Ideally, its mechanical properties should match the native spinal cord, *i.e.* 0.8–1.37 MPa Young’s Modulus [6, 7]. Other material properties of critical importance include swellability, degradability and biocompatibility.

Another challenge to address when designing TE scaffolds is to tailor the material to induce a desired immunomodulatory effect. Hyaluronic acid (HA) is biocompatible linear glycosaminoglycan which is naturally found as a component in the extracellular matrix (ECM). HA has been used for various TE strategies, but in the case of SCI it has been shown to reduce the inflammatory response at the site of injury, as well as improve neuronal growth [8, 9]. High molecular weight (HMW-HA) in particular has been found to induce a myriad of events by inhibiting the pro-inflammatory mediators [10]. In the case of SCI, the presence of HMW-HA cascades further to reduce astrocyte reactivity around the lesion site and thus reduce the presence of the glial scar, as well as decrease the numbers of macrophages and microglia present [8, 9, 11].

Recently, a new trend in TE scaffolding materials for SCI has emerged in the form of conductivity [12]. Various conductive additives have been combined with hydrogels to increase electroconductivity. Carbon-based additives include carbon nanotubes [13, 14], carbon nanofibers [15, 16] or graphene [17, 18]. Nevertheless, the potential toxicity of the carbon-based additives limits the volume for safe doping levels in scaffolds, and this can lead to poor conductivity due to percolations issues [19, 20]. Increasing the conductivity of the material can also be done by means of chemical additives. For example, polyaniline (PANI) and polypyrrole (PPy) are frequently used for this purpose [21, 22], though the major drawbacks of using these conductive polymers arise from low conductivity in neutral pH, insolubility in water and poor biocompatibility [23–25].

A commercially available conductive polymer poly(3,4-ethylenedioxythiophene) polystyrene sulfonate (PEDOT:PSS) has been recently used for TE repair strategies [26–28], however it has many serious limitations. For instance, due to its chemical structure, PEDOT:PSS can only be utilised in its current form. As it is hydrophilic, the water solubility of PEDOT:PSS is dependent on the presence of the PSS component, thus limiting the use of PEDOT when creating various hydrogel specifications. The conductivity of the polymer is also lower due to the dependence on the presence of the insulating PSS component, which possesses poor biofunctionality and biocompatibility properties [29, 30].

To overcome this, we synthetise PEDOT nanoparticles (NPs) by means of a chemical oxidation polymerization in miniemulsion. The disperse phase of the ethylenedioxythiophene (EDOT) monomer is mixed by means of ultrasonication with the continuous phase, comprising of water and poly(diallyldimethylammonium chloride) (PDADMAC) as a surfactant, forming small nano-droplets which are stabilized by the surfactant. The addition of iron(III) p-toluenesulfonate hexahydrate starts the reaction, with the nano-droplets acting as nanoreactors, thus creating conductive PEDOT NPs. The utilisation of NPs created using the outlined method instead of the commercial PEDOT:PSS to create a conductive polymer allows to not only increase the possible variability in the hydrogel components used to create the scaffold, but also for variability in the NPs surface functionalisation by altering the surfactant used during the synthesis.

In addition, a TE scaffold comprising of electro-conductive PEDOT NPs, gelatin and hyaluronic acid (gel:HA:PEDOT-NPs) has been developed for SCI applications. The developed gel:HA:PEDOT-NPs scaffold was fully characterised in terms of morphological, mechanical, electro-conductive, chemical and biocompatibility responses. A complete study of the structure/property/function relationships of carefully tailored scaffolds for optimised performance at the site of injury was carried out. The gel:HA:PEDOT-NPs scaffolds show promise in promoting repair and regeneration.

## Materials and methods

### Materials

Poly(diallyldimethylammonium chloride) solution (PDADMAC) 20 wt.% in hydrogen peroxide (H_2_O_2_), 3,4-ethylenedioxythiophene (EDOT), iron(III) p-toluenesulfonate hexahydrate (Fe-Tos), H_2_O_2_ (30 wt.%), gelatin (300 Bloom, Type A), N-(3-Dimethylaminopropyl)-N′-ethylcarbodiimide hydrochloride (EDC), N-Hydroxysuccinimide (NHS) and phosphate buffered saline (PBS) were all purchased from Sigma-Aldrich (Ireland). Hyaluronic acid (1.8 MDa molecular weight) was purchased from Shanghai Easier Industrial Development (China).

#### Synthesis of PEDOT Nanoparticles (PEDOT NPs)

PEDOT nanoparticles (NPs) were synthesized by means of chemical oxidation polymerization in miniemulsion. Briefly, PDADMAC aqueous solution was diluted in 40 mL of DI water, followed by the addition of 0.037 M of EDOT. The solution was stirred at 800 rpm for 5 min and then, ultrasonicated by tip ultrasonication for 10 min over ice to obtain a miniemulsion. Addition of 10 mL of Fe-Tos solution in DI water (0.056 M) to the miniemulsion was carried out in a dropwise manner under 45 °C and constant stirring. A solution of hydrogen peroxide at 0.001 M was then added and the reaction was left to run overnight. The NPs were purified by centrifugation at 8700 rpm for 20 min, with the supernatant discarded. This was repeated three times, with final redispersion in 40 mL of DI water.

#### Preparation of gelatin/hyaluronic acid/ PEDOT NPs scaffolds

**Fig. 1:**
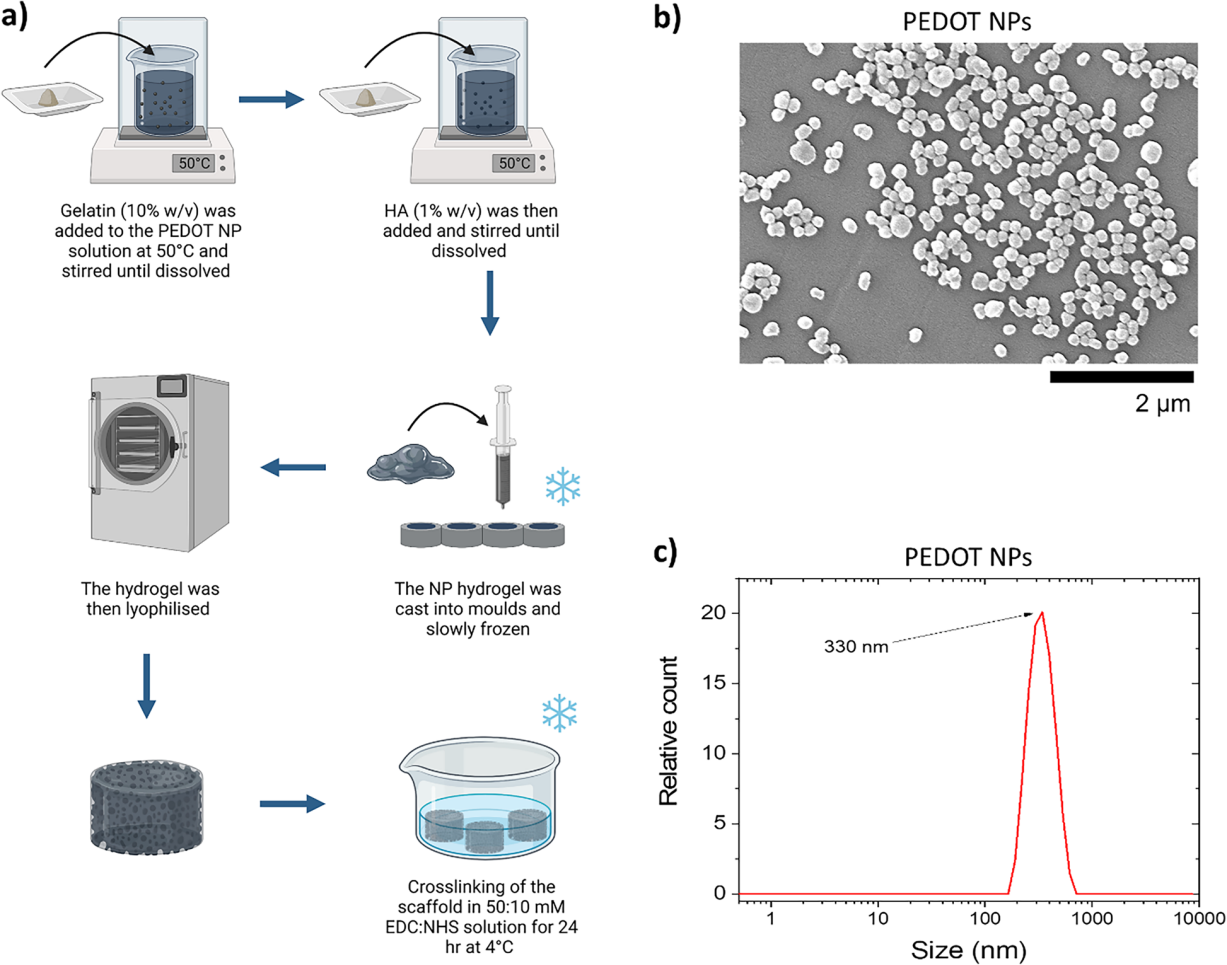
**a** Synthesis schematic of gel:HA:PEDOT-NPs scaffolds. Incorporation of PEDOT NPs into Gel:HA hydrogel and processing by means of lyophilisation develops porous scaffolds, (image created with BioRender.com). **b** SEM images of synthesized PEDOT NPs. **c** DLS measurement of PEDOT NPs in the hydrodynamic state

To prepare the gel:HA:PEDOT-NPs hydrogel solution, 10% *w/v* of gelatin was added to NPs solution synthesised as described above at 50 °C and stirred until fully dissolved. 1% *w/v* of HA was subsequently added to the solution and stirred until dissolved. To obtain different concentrations of the NPs, dilution of the NPs solution was conducted to create 1 × , 0.5 × and 0.25 × NPs concentrations. Control samples were synthesized in a similar manner, using DI water instead of NPs solution. The gel:HA:PEDOT-NPs hydrogel solutions were poured into circular moulds, 13.5 mm in diameter and 4 mm high and slowly frozen to –20 °C for 2 h, followed by – 80 °C overnight. The cast hydrogels were lyophilised with Eurotherm LS40/60, (Severn Science Ltd, Bristol, England) with cooling for 8 h at − 30 °C at 100 mbar, first drying for 16 h at − 10 °C under 0.1 mbar and secondary drying for 2 h at 20 °C (under vacuum). The scaffolds with different composition (Table 1) were then removed from the moulds and crosslinked with 50 mM:10 mM EDC:NHS in 1 × PBS solution at 4 °C for 24 h, before washing trice in DI water. The scaffolds preparation schematic is shown in Fig. 1 (a).

**Table 1:** Composition of the prepared gel:HA:PEDOT-NPs scaffolds

**Sample Name**	**PEDOT Concentration (mg/ml)**	**Gelatin (% *w/v*)**	**Hyaluronic Acid (% *w/v*)**
gel:HA:PEDOT-NPs 1 ×	2.6	10	1
gel:HA:PEDOT-NPs 0.5 ×	1.3	10	1
gel:HA:PEDOT-NPs 0.25 ×	0.65	10	1
Gel:HA Control	N/A	10	1

#### Characterisation of gelatin/hyaluronic acid/ PEDOT NPs scaffolds

Synthesized NPs were visualised via SEM analysis with Hitachi SU70 SEM at an imaging voltage of 10 kV. The NPs solution was added dropwise onto a glass slide and the water was allowed to evaporate. Lyophilised gel:HA:PEDOT-NPs scaffolds were coated with gold sputtering before the SEM analysis. NP analysis was also conducted with Dynamic light scattering (DLS). Particle size of the PEDOT NPs is measured using a particle size analyzer (Zetasizer Nano ZS, Malvern Instruments, Malvern, UK). NPs were diluted to low concentration in a 0.3 M KCl aqueous solution.

Compression tests were conducted on gel:HA:PEDOT-NPs scaffolds using an in-house compression test facility equipped with a 1 kN load cell and compressed at a rate of 1 mm/s between parallel plates. The Young’s Modulus of the samples was calculated as the slope in the linear region of a normalised stress vs. strain graph, as shown in Supplementary Figure S1.

Dielectric measurements in the frequency range from 5 × 10^–2^ to 3 × 10^6^ Hz were performed using a Novocontrol Broadband Dielectric Spectrometer (Hundsagen, Germany) consisting of an Alpha Analyzer. The measurements were performed in N_2_ atmosphere at 37 °C using the temperature control system of a Novocontrol Quatro cryosystem, with an accuracy of ± 0.1 °C during each sweep in frequency. Moulded disc shaped samples of about 3–4 mm thickness and 10 mm diameter were used. The experimental uncertainty was less than 5% in all cases. Measurements were made both with non-crosslinked samples and with crosslinked samples, the latter both dry and re-hydrated in PBS. Measurements were carried out with an excitation voltage of 1 V. Note that for each composition five measurements were made.

Spectra of gel:HA:PEDOT-NPs scaffolds were obtained utilising a Spectrum 100 FTIR (PerkinElmer, USA) in the range 500–4000 cm^−1^ for 10 scans.

Prior to swelling tests, gel:HA:PEDOT-NPs scaffolds were dried in a vacuum oven overnight, weighed and then placed in PBS at 37 °C for a period of up to 96 h. Subsequently, the wet weight of the samples was measured, and the swelling degree calculated as follows:

1 $$\left(\frac{Ws-Wd}{Wd}\right)*100\%$$

where, *Ws* is the hydrogel swollen mass, and *Wd* is the dried mass.

Rheological properties of the gel:HA:PEDOT-NPs hydrogel prior to scaffold lyophilisation were analysed with a hybrid rheometer (TA Instruments, USA), using techniques described in [15, 31]. Briefly, disposable 25 mm aluminium rheological plates were used for the analysis with a measurement gap of 550 µm. The gel:HA:PEDOT-NPs hydrogels were tested using the following regimes: 1) strain sweeps ranging from 0.1–100% at 1.59 Hz to determine the viscoelastic range of the samples, 2) frequency sweeps of 0.1–100 rad/s at the determined constant strain of 2%, 3) steady state flow tests with shear rates ranging from 0.5–500 s^−1^, and 4) recovery test under shear rates of 0.1 s^−1^ and 100 s^−1^. All the experiments were conducted at 37 °C.

### Preliminary biocompatibility of gelatin/hyaluronic acid/ PEDOT NPs scaffolds

#### In-vitro studies

Mesenchymal Stem Cells (MSCs) were grown in Minimum Essential Medium alpha (MEMα) supplemented with 20% foetal bovine serum, 1% GlutaMAX and 1% Penicillin–Streptomycin, (all materials purchased from Sigma Aldrich (USA)), in a 5% CO_2_ environment.

PEDOT NPs were first tested for their cytotoxicity with MSCs seeded into a 96 well-plate at a density of 0.025 × 10^6^ cells/well. PEDOT NPs in the following concentrations of 1 × , 0.5 × and 0.25 × were added to the wells and cultured over a period of 96 h. Alamar Blue*™* (Invitrogen, Thermofisher, USA) was added at 10% of the volume of the well and incubated for 5 h. Measurement of the cell fluorescent emission was carried out with Cytation 5 (BioTek, Agilent Technologies, USA) at wavelength 540/590 nm. Cells incubated without the presence of the PEDOT NPs acted as controls.

Cells were seeded onto the pre-conditioned gel:HA:PEDOT-NPs scaffolds at a density of 0.2 × 10^6^ cells/construct in a 24-well plate, supplemented with 1 mL of cell culture media and incubated overnight. For cell cytotoxicity evaluation, Alamar Blue*™* protocol was used following the manufacture instructions, and measurement was carried out as outlined above. Cells incubated without the presence of scaffolds acted as a control.

To assess the cytocompatibility of the gel:HA:PEDOT-NPs scaffolds, MSCs were seeded onto the scaffolds at a density of 0.2 × 10^6^ cells/construct in a 24-well plate and cultured for a period of 96 h, followed by staining with Calcein AM (Invitrogen, Thermofisher, USA) and propidium iodine (Sigma Aldrich, USA) and imaged by means of Carl Zeiss Microscope (USA) for fluorescence imaging. Quantitative analysis of the cell viability was completed using the ImageJ software.

To visualise the morphology and cell attachment to the gel:HA:PEDOT-NPs scaffolds, Vybrant DiI (1:200) (Invitrogen, Thermofisher, USA) stained MSCs were seeded onto the scaffolds at a density of 0.1 × 10^6^ cells/construct in a 24-well plate, following the protocol outlined above and cultured for a period of 96 h. The cells were then fixed with 4% paraformaldehyde (PFA), stained with DAPI (1:1000) (Sigma Aldrich, USA) and imaged.

#### Preliminary in-vivo spinal cord injury model

NIH guidelines for laboratory animal care and safety were followed throughout the experiment. Spinal cord injury and scaffold implant were performed as previously described [32]. Briefly, Male Fisher 344 rats with an average weight of 500 gr were deeply anesthetised by means of IM injection of ketamine/domitor (75 mg/kg and 0.5 mg/kg, respectively). Laminectomy at the T2-T4 level was performed following by opening of the dura. The spinal cord was transacted at the T3 level using a combination of micro-scissors and micro-aspiration, forming a 2 mm cavity. A 2 mm gel:HA:PEDOT-NPs 1 × scaffold was carefully inserted into the lesion site, followed by suturing of two muscle layers *(n* = *4)*. The control also underwent a SCI procedure without the implementation of the scaffold *(n* = *1)*.

Following the surgery, the animals were housed separately on a 12:12 h light/dark cycle, with ad libitum access to food and water. Administration of antibiotic injection was done once daily for seven days. Bladders were manually emptied by gentle massage twice a day until partially autonomous urination was restored.

#### Immunohistochemistry

Four weeks post scaffold implantation, animals were perfused transcardially by means of 4% PFA in 0.1 M PBS (pH 7.4) at 4 °C. The spinal cord tissue was collected and stored in 4% PFA overnight at 4 °C, followed by immersion with 30% sucrose in 0.1 M PBS at 4 °C for an additional 48 h and stored at 4 °C until use, prior to cryosectioning (Leica). The spinal segment of interest was imbedded in Tissue Plus Optimum Cutting Temperature compound (Scigen, Thermo Fisher, USA), sectioned into 35 µm thick sections and mounted on slides.

The sectioned slides were first blocked by means of 5% donkey serum, followed by incubation with primary antibodies overnight at 4 °C as follows: mouse-anti-NF200 (1:500, Millipore, USA), chicken-anti-GFAP (1:500, EnCore Biotechnology, USA), mouse-anti-ED1 (1:500, Biorad, USA), goat-anti-IBA1 (1:100, Abcam, USA). After washes with Tris–HCl Buffered Saline (TBS), sectioned slides were incubated with secondary antibodies overnight at 4 °C as follows: donkey-anti-mouse Alexa Fluor 488, donkey-anti-chicken Alexa Fluor 647 or donkey-anti-goat Alexa Fluor 568 (1:250, Invitrogen, USA). Sections were washed with TBS and cover slipped with Fluoromount G (Southern Biotechnology Associates, USA). The stained spinal sections were imaged by means of BZ-X710 fluorescent microscope (Keyence, USA). Quantitative analysis of the histological slides was completed using the ImageJ software.

#### Statistical analysis

Experiments were conducted in triplicates, with the data presented as mean ± standard deviation. To determine the statistical significance, one-way analysis of variance (ANOVA) was employed with the *p*-value of < 0.05 considered as statistically significant (*, *p* < 0.05). A two-way ANOVA was employed for the Alamar Blue*™* cytocompatibility analysis, with the *p*-value of < 0.05 considered as statistically significant (*, *p* < 0.05).

## Results

### Morphological characterisation of PEDOT nanoparticles

The PEDOT NPs were synthesized successfully and incorporated into Gel:HA gels as shown in Fig. 1 (a) to develop gel:HA:PEDOT-NPs scaffolds. Analysis of the PEDOT NPs by means of SEM showed a high number of NPs with a stable, round morphology, as shown in Fig. 1 (b). ImageJ analysis of the images indicated that the NPs have a diameter of 187.3 ± 20.2 nm while DLS analysis showed a hydrodynamic size of the PEDOT NPs to be 330 nm, shown in Fig. 1 (c). Hydrodynamic values are typically higher than the ones measured utilising microscopy images due to swelling and aggradation factors.

### Morphological characterisation of PEDOT scaffolds

**Fig. 2:**
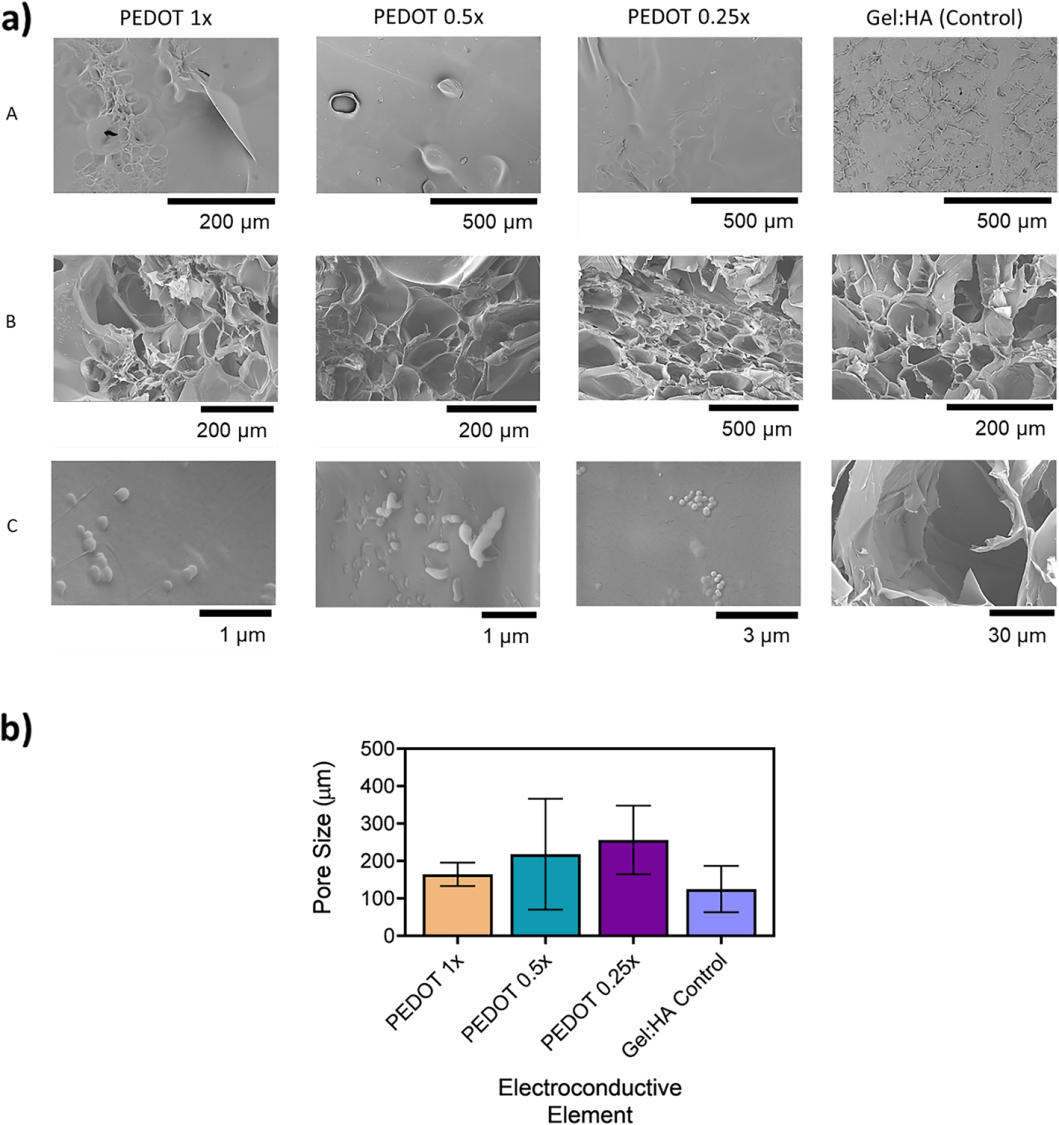
**a** SEM images of lyophilised gel:HA:PEDOT-NPs scaffolds with different PEDOT NPs concentrations (1 × , 0.5 × , 0.25 × and Gel:HA control with no NPs). A-Surface view of gel:HA:PEDOT-NPs scaffolds. B-Internal Porosity. C-Magnification of PEDOT NPs on the internal surfaces of the gel:HA:PEDOT-NPs scaffolds. **b** Internal pore diameter of gel:HA:PEDOT-NPs scaffolds and Gel:HA control scaffolds, * *p* < 0.05, (*n* = 3, mean ± SD) is indicated where statistical difference is observed

The morphology of the scaffolds was analysed by means of SEM imaging and is shown in Fig. 2 (a). ImageJ analysis of the scaffold porosity shows that the average pore size ranges from 164.5 ± 31.3 µm for PEDOT 1 × , 218.2 ± 147.9 µm for PEDOT 0.5 × to 256.1 ± 91.7 µm for the PEDOT 0.25 × , indicated in Fig. 2 (b). Pore diameter of the control samples with no NPs were measured at 124.9 ± 61.8 µm. No statistical difference between the groups was found. PEDOT NPs were dispersed homogenously throughout the scaffold structure in all of the gel:HA:PEDOT-NPs scaffolds, as seen in Fig. 2 (a), row C. The NPs were both embedded into the material, as well as present in the struts of the pores for all PEDOT NP concentrations.

### Mechanical characterisation of PEDOT scaffolds

**Fig. 3:**
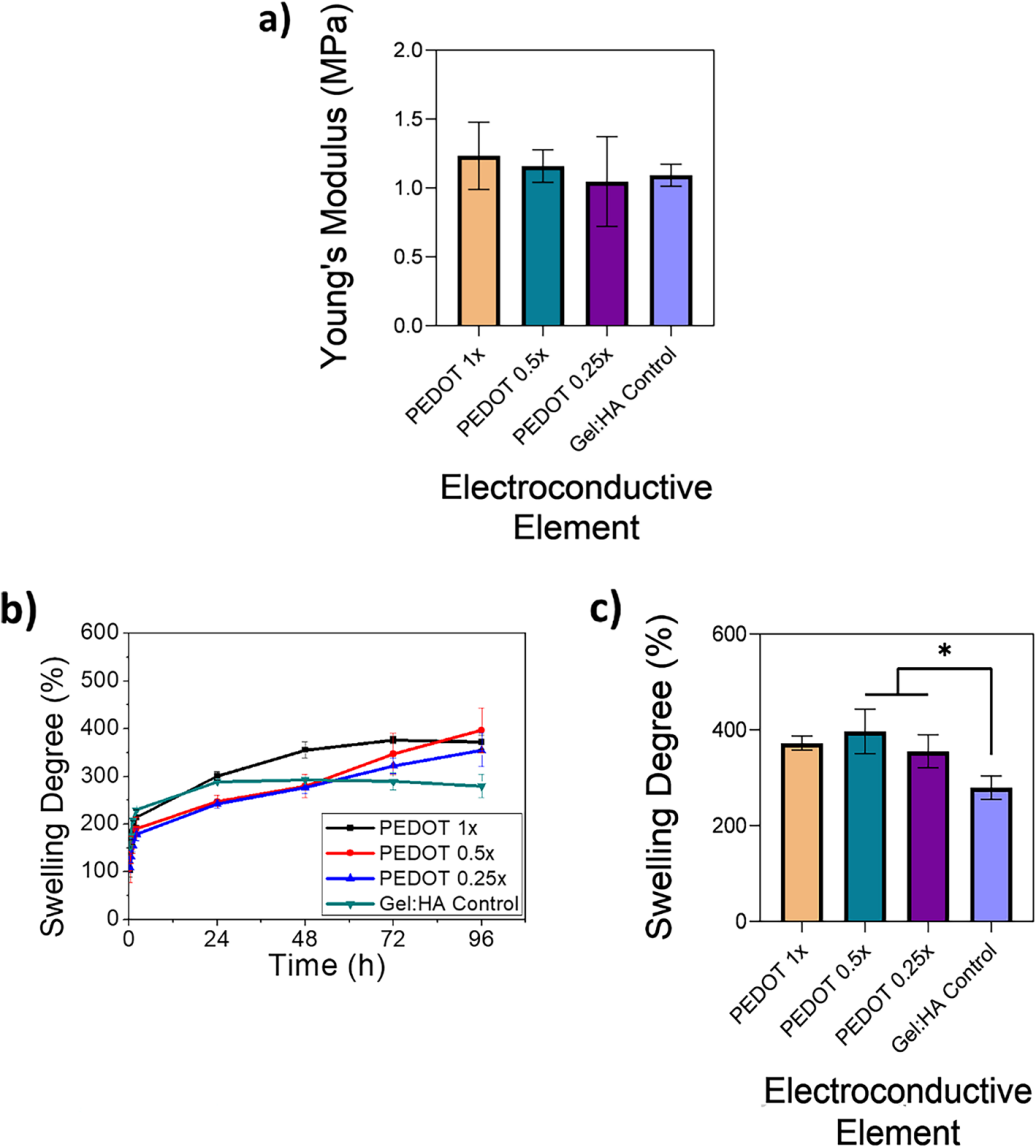
**a** Young’s Modulus of the gel:HA:PEDOT-NPs scaffolds and Gel:HA control scaffolds, * *p* < 0.05, (*n* = 3, mean ± SD) is indicated where statistical difference is observed. **b** Swelling profile of gel:HA:PEDOT-NPs scaffolds and Gel:HA control scaffolds over a period of 96 h. **c** Swelling degree of gel:HA:PEDOT-NPs scaffolds and Gel:HA control scaffolds at the 96 h timepoint scaffolds, * *p* < 0.05, (*n* = 3 samples, mean ± SD) is indicated where statistical difference is observed

Unconfined compression tests were conducted on the gel:HA:PEDOT-NPs scaffolds to determine their mechanical properties (Fig. 3 (a)). The Young’s Modulus was not significantly affected by the presence of the PEDOT NPs, though increasing the concentration of the NP increased the Young’s Modulus from 1.05 ± 0.3 MPa for the PEDOT 0.25 × samples to 1.2 ± 0.2 MPa for the PEDOT 1 × samples. When comparing the control of Gel:HA to the PEDOT 1 × sample, the control measures 1.09 ± 0.08 MPa in the Young’s Modulus. No statistical difference between the groups was found.

### Swelling degree of PEDOT scaffolds

The swelling degree of the gel:HA:PEDOT-NPs scaffolds was conducted over a period of 96 h to determine the effectiveness of the crosslinking regime, as well as the degradation rate, and is shown in Fig. 3 (b). Moisture uptake by the dried-out scaffolds was the most prominent during the first 48 h, with a steady rise in the swelling degree observed for all PEDOT NP samples throughout the 96 h observation time as the water equilibrium content is reached. The presence of the NPs appears to affect the crosslinking regime to a small degree, though the swelling degrees does not vary in a statistically significant manner between the different NP concentrations at the end time point, as shown in Fig. 3 (c).

### Conductive characterisation of PEDOT scaffolds

**Fig. 4:**
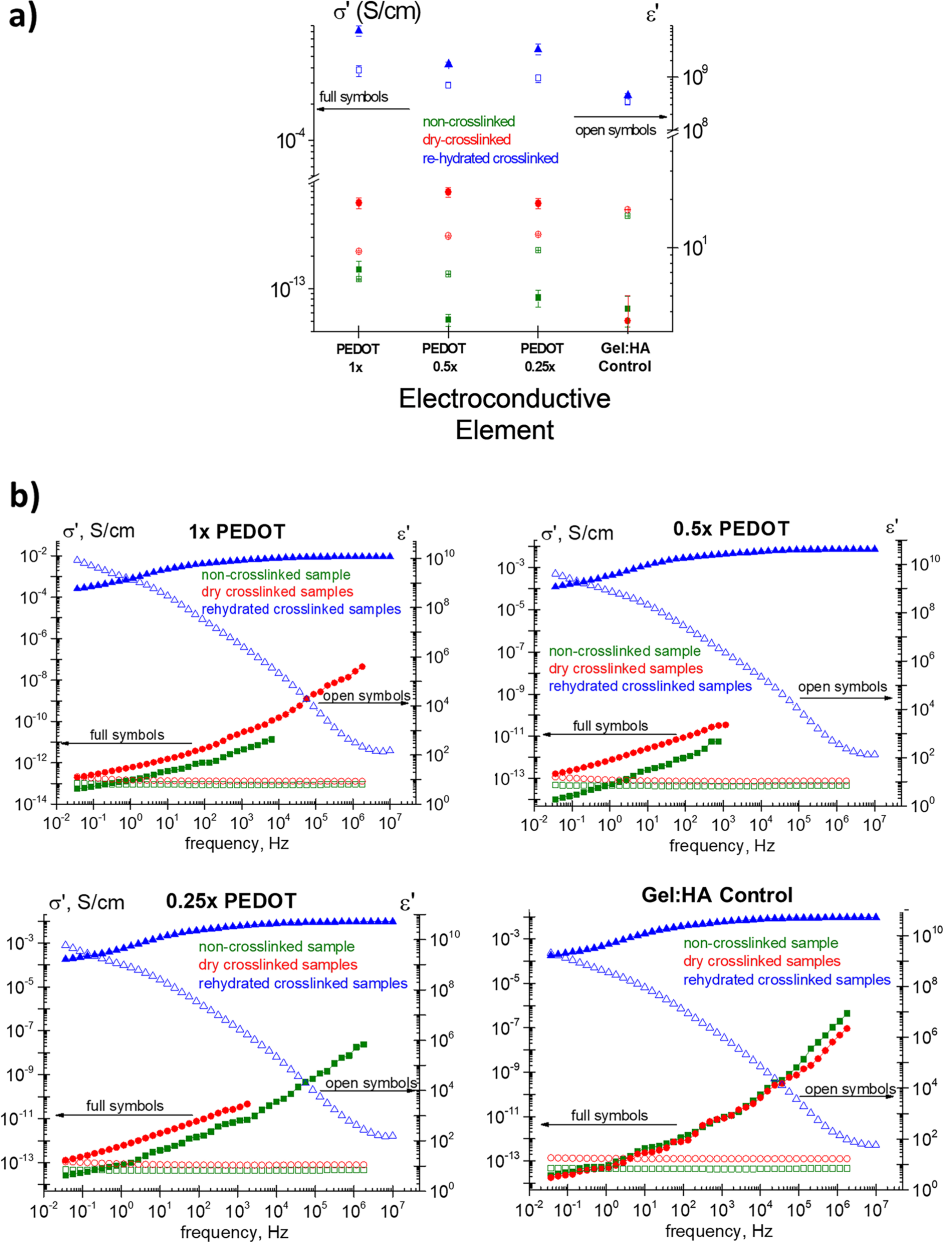
**a** Conductivity of the gel:HA:PEDOT-NPs scaffolds and Gel:HA control scaffolds in the non-crosslinked form, as well as crosslinked form, both in their dry and re-hydrated state. **b** Frequency dependence of the conductivity and of the dielectric permittivity at 37ºC for non-crosslinked samples, as well as dry and rehydrated crosslinked the gel:HA:PEDOT-NPs samples

To study the contribution of the PEDOT NPs on the conductivity of the scaffold material, dielectric measurements were conducted. The measured dielectric permittivity and AC conductivity for non-crosslinked samples, and for dry and rehydrated crosslinked samples at 37 °C and at 1 Hz, are shown in Fig. 4 (a) and in Table 2. Conductivity increases as function of PEDOT concentration in both the non-crosslinked samples and the rehydrated samples. For the dried samples the electrical conductivity of the scaffolds clearly shows values below the percolation threshold which lead to similar electrical conductivity values at different NPs concentrations. This trend is clearly evident in crosslinked samples with an electrical conductivity of 6.2 × 10^–13^, 7.9 × 10^–13^, 6.2 × 10^–13^ S/cm at 0.25 × , 0.5 × and 1 × PEDOT NPs. A similar trend is observed for non-crosslinked samples with a slight increase for the 1 × PEDOT NPs. Figure 4 (b) shows the frequency dependence of the dielectric permittivity and the AC conductivity measured for non-crosslinked, as well as dry and rehydrated crosslinked samples at 37 ºC. Dielectric permittivity decreases with the increasing frequency and the conductivity increases with increasing frequency.

**Table 2:** Electrophysical characteristic of the gel:HA:PEDOT-NPs scaffolds at a field frequency of 1 Hz measured at 37ºC

	***Sample***	***Conductivity (S/cm)***	***Permittivity***
***Non-crosslinked***	PEDOT 1 ×	1.5 × 10^–13^ ± 3.1 × 10^–14^	6.4 ± 0.03
	PEDOT 0.5 ×	5.2 × 10^–14^ ± 6.7 × 10^–15^	6.85 ± 0.02
	PEDOT 0.25 ×	8.3 × 10^–14^ ± 1.5 × 10^–14^	9.65 ± 0.03
	Gel:HA Control	6.5 × 10^–14^ ± 2.1 × 10^–14^	15.83 ± 0.04
***Crosslinked (dry)***	PEDOT 1 ×	6.3 × 10^–13^ ± 6.9 × 10^–14^	9.5 ± 0.07
	PEDOT 0.5 ×	7.9 × 10^–13^ ± 8 × 10^–14^	11.8 ± 0.1
	PEDOT 0.25 ×	6.2 × 10^–13^ ± 6.8 × 10^–14^	12.1 ± 0.08
	Gel:HA Control	1.6 × 10^–13^ ± 3.7 × 10^–14^	17.3 ± 0.04
***Crosslinked (hydrated)***	PEDOT 1 ×	8.3 × 10^–4^ ± 8.1 × 10^–5^	1.34 × 10^9^ ± 3.09 × 10^8^
	PEDOT 0.5 ×	4.3 × 10^–4^ ± 2.05 × 10^–5^	7.05 × 10^8^ ± 8.6 × 10^7^
	PEDOT 0.25 ×	5.8 × 10^–4^ ± 5.9 × 10^–5^	9.5 × 10^8^ ± 1.6 × 10^8^
	Gel:HA Control	2.3 × 10^–4^ ± 3.4 × 10^–5^	3.5 × 10^8^ ± 4.7 × 10^7^

### Chemical analysis of PEDOT scaffolds

**Fig. 5:**
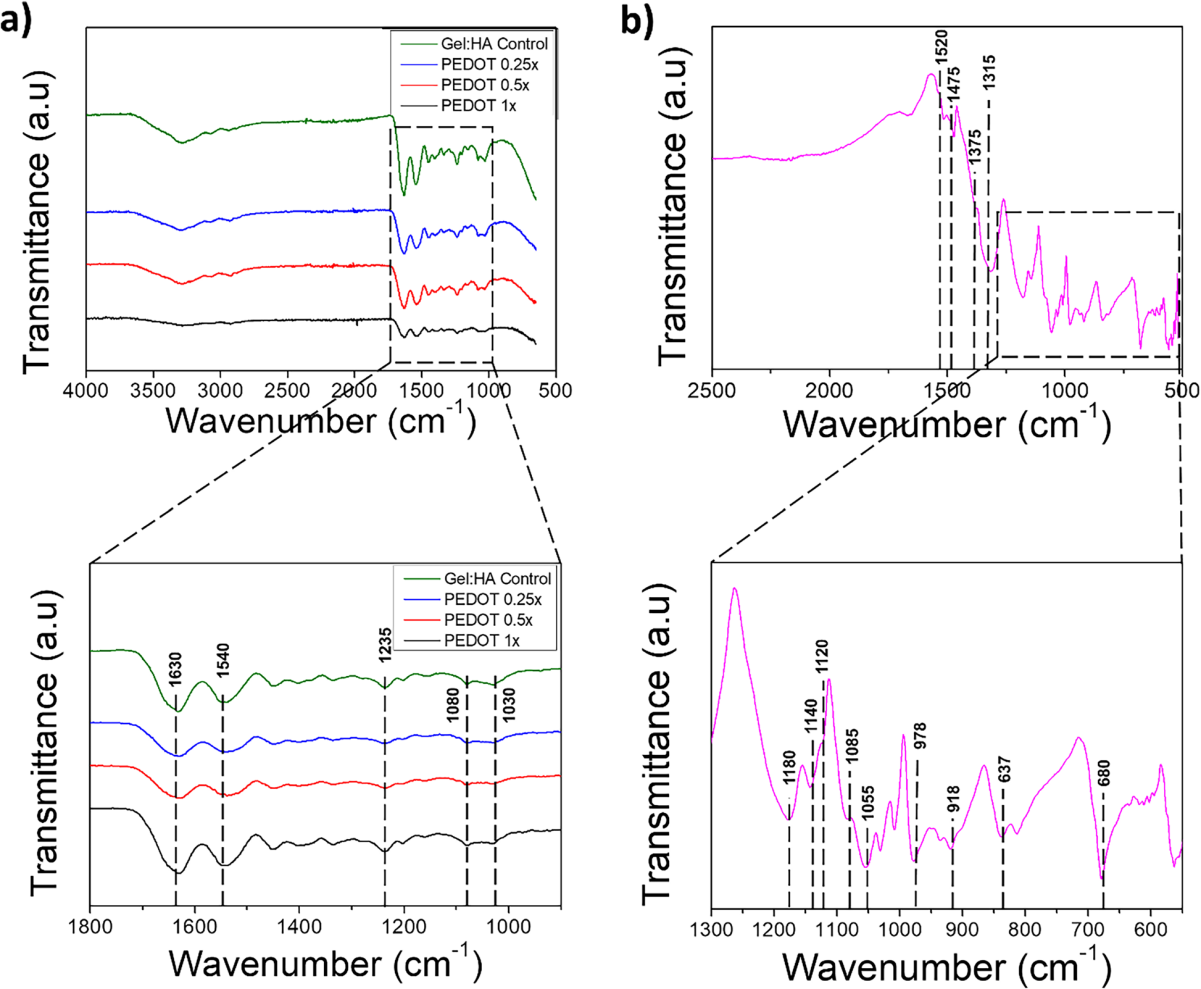
**a** FTIR spectra of gel:HA:PEDOT-NPs scaffolds and Gel:HA control scaffolds. **b** FTIR spectra of PEDOT NPs

FTIR analysis of the gel:HA:PEDOT-NPs scaffolds is shown in Fig. 5 (a). A wide characteristic band occurs at 3300 cm^−1^ relating to the O–H and N–H stretching attributed to the presence of both gelatin and HA components in the scaffold composition [15, 33, 34], with the gelatin amide A and B bands observed at 3500–3200 cm^−1^ and 3070 cm^−1^, respectively [35]. The next prominent HA band occurs at 2940 cm^−1^ and is attributed to C-H stretching vibration [33].

Characteristic absorption bands relating to the gelatin component in the scaffolds occur at 1630 cm^−1^ representing the amide I band, which relates to the C-O and C-N stretching originating from the -NH group of the gelatin. At the same absorption band, the amide carbonyl attributed to HA is also present [15, 33, 34]. Gelatin amide II and III bands are observed at 1540 cm^−1^ and 1235 cm^−1^ respectively, attributed to C-N stretching and N–H bending of the gelatin component in the scaffold [15, 34]. The absorption band at 1030 cm^−1^ informs of the presence of linkage stretching of C–OH of the HA scaffold component [33].

Absorption peaks associated with the NPs are overshadowed by the more prominent peaks of gelatin and HA. However, a small absorption peak at 1080 cm^−1^ can be attributed to the C–O–C bending vibration in the ethylenedioxy group, potentially identifying the presence of PEDOT NPs [36].

The spectra of PEDOT NPs only with their characteristic bands is shown in Fig. 5 (b). The peaks at 1520 cm^−1^ and 1475 cm^−1^ are attributed to the asymmetric stretching vibration of C = C, the peak at 1375 cm^−1^ to the C–C and C = C stretching of the thiophane ring and the peak at 1315 cm^−1^ to the inter-ring stretching of C–C. The peaks present at 1085, 1180, 1155, 1040 cm^−1^ are attributed to the C–O–C bending vibration in ethylenedioxy group and the peak at 1120 cm^−1^ to the C–O–C band stretching vibration. The characteristic bands at 978, 918, 837, 680 cm^−1^ are assigned to the stretching vibrations of the C-S-C bond in thiophene ring [36–38].

### Rheological analysis of PEDOT hydrogel

**Fig. 6:**
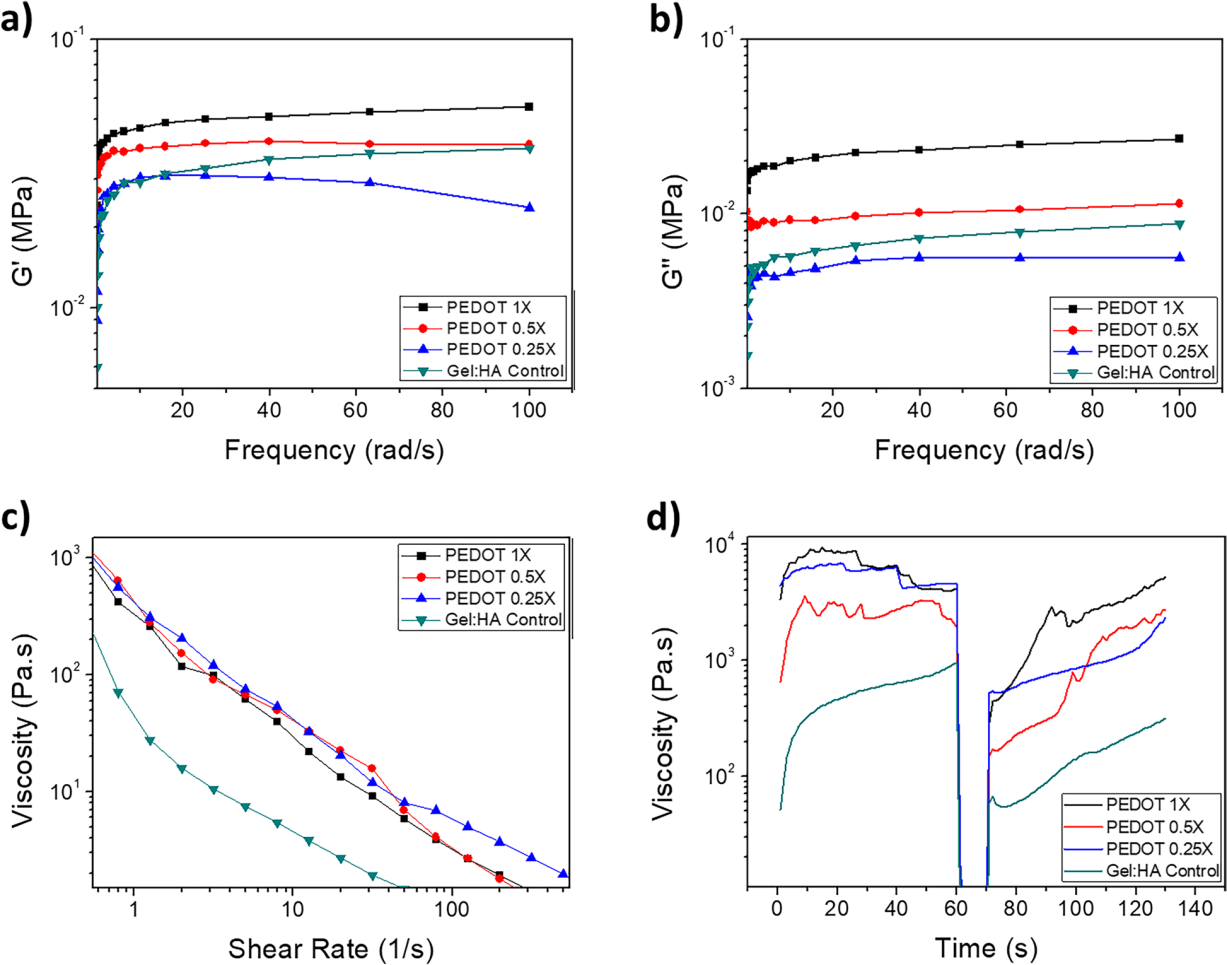
Rheological analysis of gel:HA:PEDOT-NPs hydrogels and Gel:HA control hydrogel at different PEDOT NPs concentrations. **a** Storage modulus as a function of frequency **b** Loss modulus as a function of frequency **c** Viscosity dependent on shear rate and **d** Recovery profile simulating 3D printing with viscosity depended on shear stress

The storage modulus and the loss modulus increase with an increase in frequency and are shown in Fig. 6 (a) and (b). The storage modulus remained higher than the loss modulus for all the hydrogel samples, indicating that the hydrogels are within the elastic, solid-like response.

When a shear force is applied to the PEDOT hydrogels, the viscosity decreases with an increase in shear, exhibiting a typical shear-thinning behaviour, as shown in Fig. 6 (c). At lower shear rates of 2 s^−1^ the viscosity of the PEDOT 1 × hydrogel was 117. 8 Pa.s, while at higher shear rates it decreased to 5.8 Pa.s at 50 s^−1^. Viscosity of the control sample followed the same trend, 15.9 Pa.s at 2 s^−1^ decreasing to 1.6 Pa.s at 50 s^−1^. All other samples behaved similarly with higher NPs concentrations slightly increasing viscosity.

Recovery tests of the un-crosslinked PEDOT NP hydrogel samples were also conducted to investigate their potential to be used in 3D printing scenarios and are shown in Fig. 6 (d). The addition of PEDOT NPs into the Gel:HA material improved the hydrogel’s recovery profile, though overall, all samples showed a sharp decrease in the viscosity with the application of the high shear stress (0–60 s), with an instant increase in the viscosity once the shear force was removed (70–130 s).

### Preliminary in-vitro cytocompatibility assessment of PEDOT NPs

**Fig. 7:**
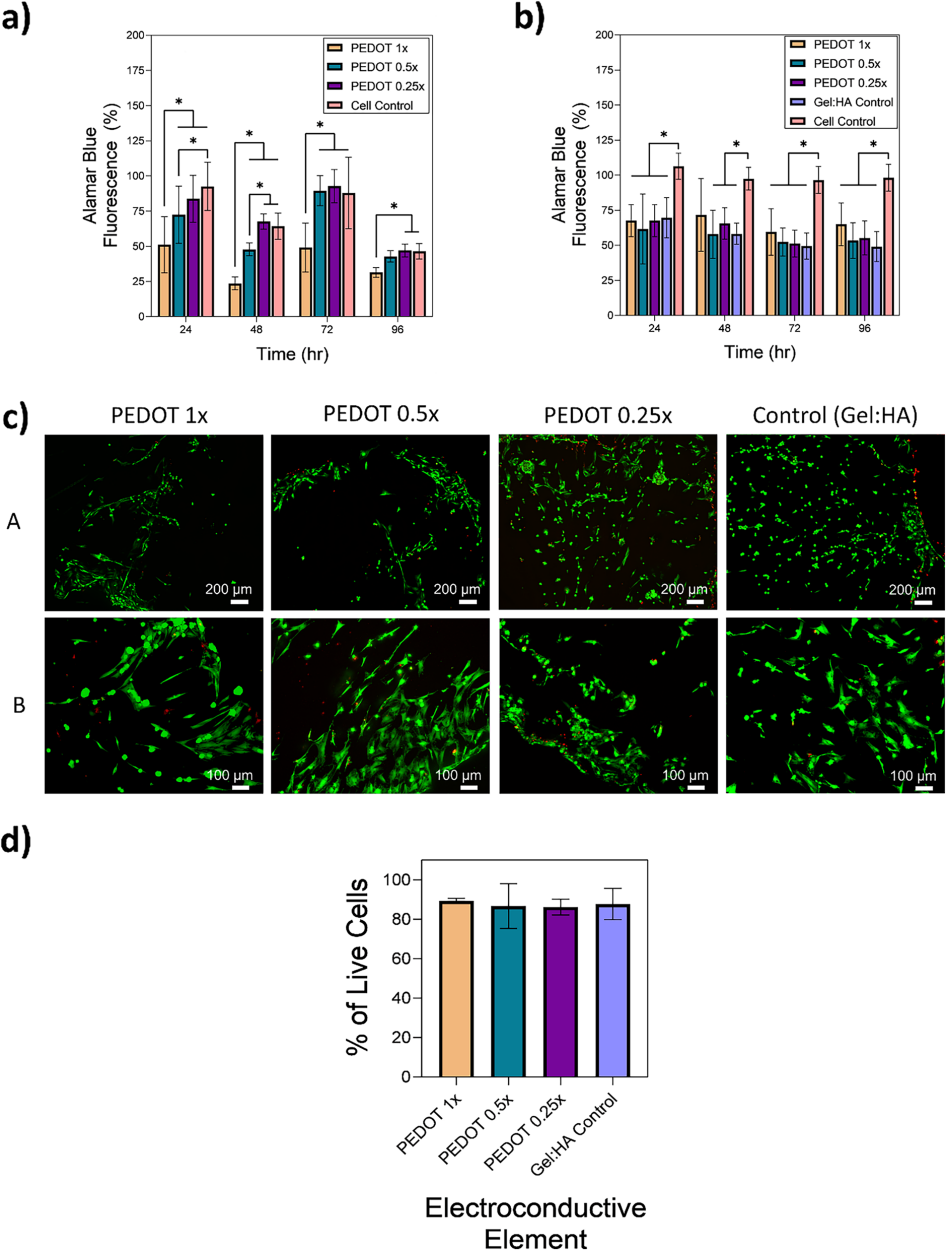
MSCs seeded on gel:HA:PEDOT-NPs scaffolds show high viability, along with spindle-like morphology typical of MSCs, when examined in-vitro. **a**-**b** Alamar Blue™ Reduction of MSCs cultured in the presence of: **a**) PEDOT NPs at different concentrations, and **b**) MSCs seeded onto the gel:HA:PEDOT-NPs scaffolds of different concentrations, both cultured for a period of 96 h, * *p* < 0.05, (*n* = 3, mean ± SD) is indicated where statistical difference is observed. **c** LIVE/DEAD staining of MSCs seeded onto gel:HA:PEDOT-NPs scaffolds of different NP concentrations and cultured for a period of 96 h. A-Top scale bar- 200 µm and B- bottom scale bar- 100 µm. **d** Quantification of live cells, gathered by means of ImageJ analysis of (c), scaffolds, * *p* < 0.05, (*n* = 3, mean ± SD) is indicated where statistical difference is observed

The potential cytotoxicity of the NPs was initially assessed prior to addition into Gel:HA scaffolds. MSCs were cultured in the presence of PEDOT NPs at different concentrations (1 × , 0.5 × , 0.25 ×) for a period of 96 h. The proliferation rates of the MSCs were measured via Alamar Blue*™* Assay every 24 h, with the results presented in Fig. 7 (a). Overall, the proliferation profiles for all samples followed a similar trend, with an increase in the proliferation occurring over the initial 72 h time point and dropping at the 96 h timepoint. Decreasing the NP concentration increased the proliferation rates of the MSCs, for example at 47.1 ± 4.6% for the 0.25 × PEDOT NPs as compared to the control with no NPs at 46.4 ± 5.4% at the 96 h timepoint, though the proliferation rates of the lower concentrations (0.5 × , 0.25 ×) surpassed the proliferation rates of the control at the 48 h and 72 h timepoints. Two-way ANOVA showed a statistical difference in the proliferation rates of cells between the groups across the 96 h observation period.

### Preliminary in-vitro cytocompatibility assessment of PEDOT scaffolds

Potential cytotoxicity of scaffolds with incorporated PEDOT NPs was conducted with MSCs cultured in the presence of the gel:HA:PEDOT-NPs scaffolds and the results are shown in Fig. 7 (b). As opposed to the results of NPs only, the proliferation rates of the higher concentrations of NPs (1 ×) in the scaffolds yielded more prominent proliferation rates than the 0.5 × , 0.25 × concentrations at 65.0 ± 15.3% for the PEDOT 1 × scaffold and 55.3 ± 12.2% for the PEDOT 0.25 × scaffold at the 96 h timepoint and were similar over the 96 h observation period. When compared to the cell control the proliferation rates were lower, though the proliferation was higher in NP scaffold groups than in the no NP control group (Gel:HA), suggesting an improved proliferation ability when MSCs are cultured on the NP scaffolds. Two-way ANOVA showed a statistical difference in the proliferation rates of cells between the groups across the 96 h observation period.

The visualisation of the cytocompatibility of the gel:HA:PEDOT-NPs scaffolds was conducted by means of LIVE/DEAD staining of MSCs cultured on the scaffolds for a period of 96 h, as shown in Fig. 7 (c). Across all groups high levels of live cells were present, with elongated spindle like cellular morphology typical of MSCs observed [39, 40]. Quantification of the live cells was conducted by ImageJ analysis and is presented in Fig. 7 (d). The cell viability of all scaffold groups was higher than 86%, with the viability of the PEDOT 1 × scaffolds being the highest at 89.4 ± 1.3%, higher than the scaffold control with no NPs.

**Fig. 8:**
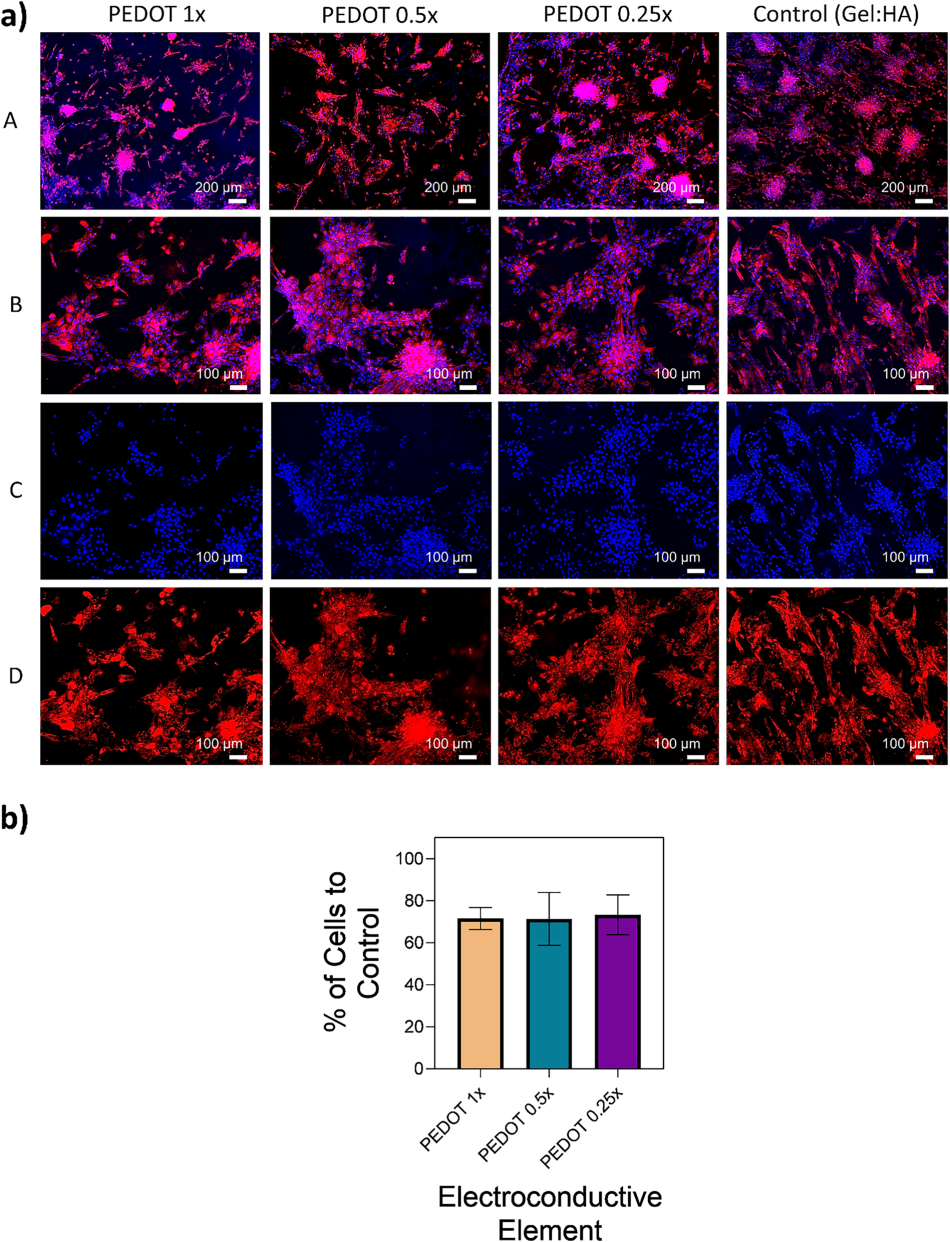
**a** MSCs seeded on gel:HA:PEDOT-NPs scaffolds show high viability, along with spindle-like morphology typical of MSCs, when examined in-vitro. MSCs previously stained with DiI (red) seeded onto gel:HA:PEDOT-NPs scaffolds of different NP concentrations and cultured for a period of 96 h, fixed and stained with DAPI (blue). (A) DiI/DAPI scale bar—200 µm, (B) DiI/DAPI scale bar—100 µm, (C) DAPI scale bar—100 µm, (D) DiI scale bar—100 µm. **b** Quantification of the number of cells when compared to the scaffold control with no NPs (Gel:HA), gathered by means of ImageJ analysis of (a), * *p* < 0.05, (*n* = 3, mean ± SD) is indicated where statistical difference is observed

To further visualise the attachment of MSCs onto gel:HA:PEDOT-NPs, previously stained DiI MSCs were seeded onto the surface of the PEDOT NP scaffolds and cultured for a period of 96 h, followed by fixation with 4% PFA and nuclear staining by means of DAPI, with the results presented in Fig. 8 (a). Quantification of the number of cells present on the scaffolds is outlined in Fig. 8 (b), taken by means of ImageJ analysis of Fig. 8 (a), using the DAPI channel to identify the number of cells present by means of cell nuclei. All PEDOT NP scaffold groups had a similar number of cells on the surface of the scaffolds, ranging from 71.4 ± 12.6% to 73.3 ± 9.5%, when compared to the number of cells present on the scaffold control with no NP (Gel:HA scaffold), though no statistical difference between the groups was found.

### Preliminary in-vivo cytocompatibility assessment of PEDOT scaffolds

**Fig. 9:**
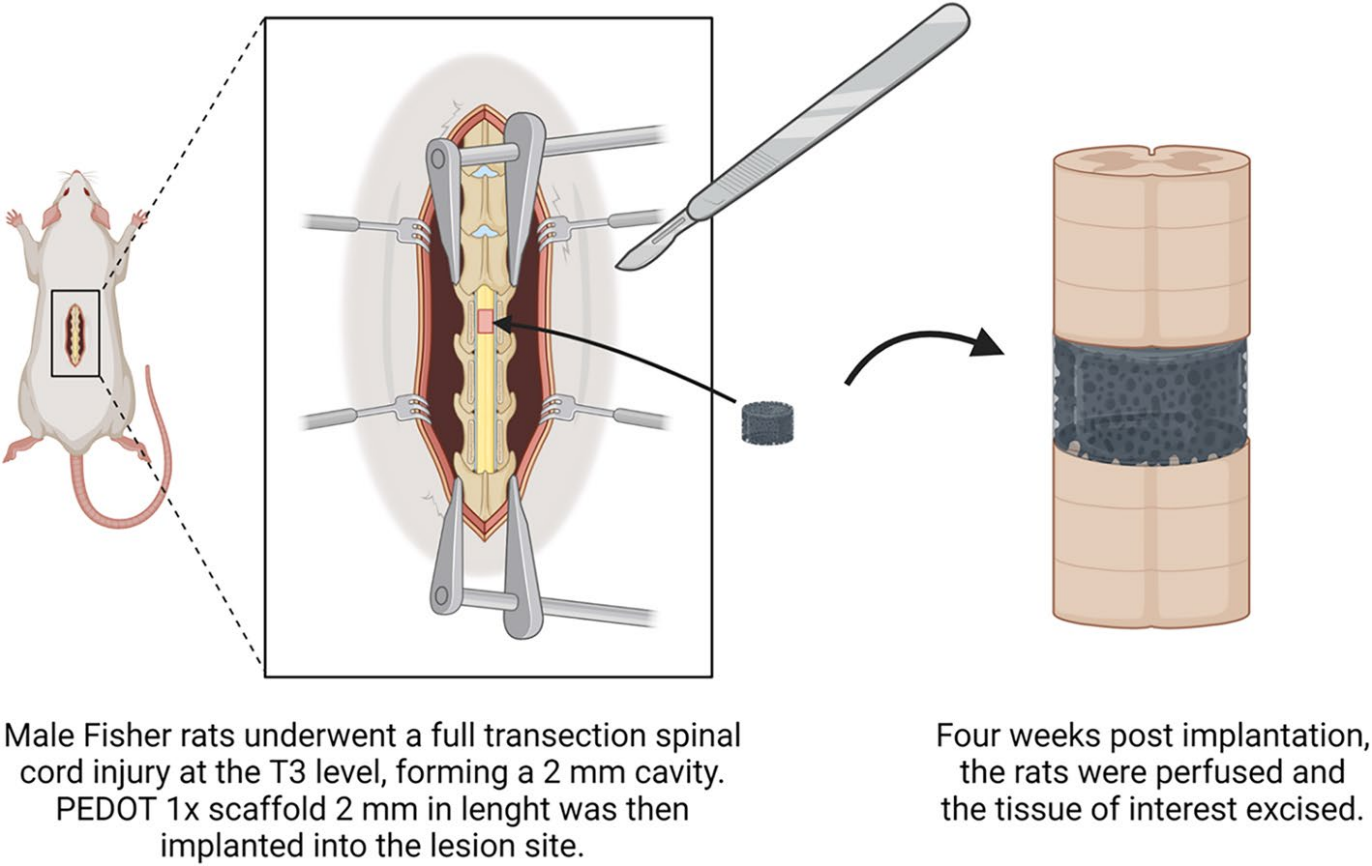
Schematic of the implementation of gel:HA:PEDOT-NPs 1 × scaffold into a 2 mm full transection rat SCI model for in-vivo assessment, (image created with BioRender.com)

In-vivo biocompatibility of the gel:HA:PEDOT-NPs 1 × scaffold was also investigated by means of implantation of the scaffold into a rat SCI lesion model, as depicted in Fig. 9.

**Fig. 10:**
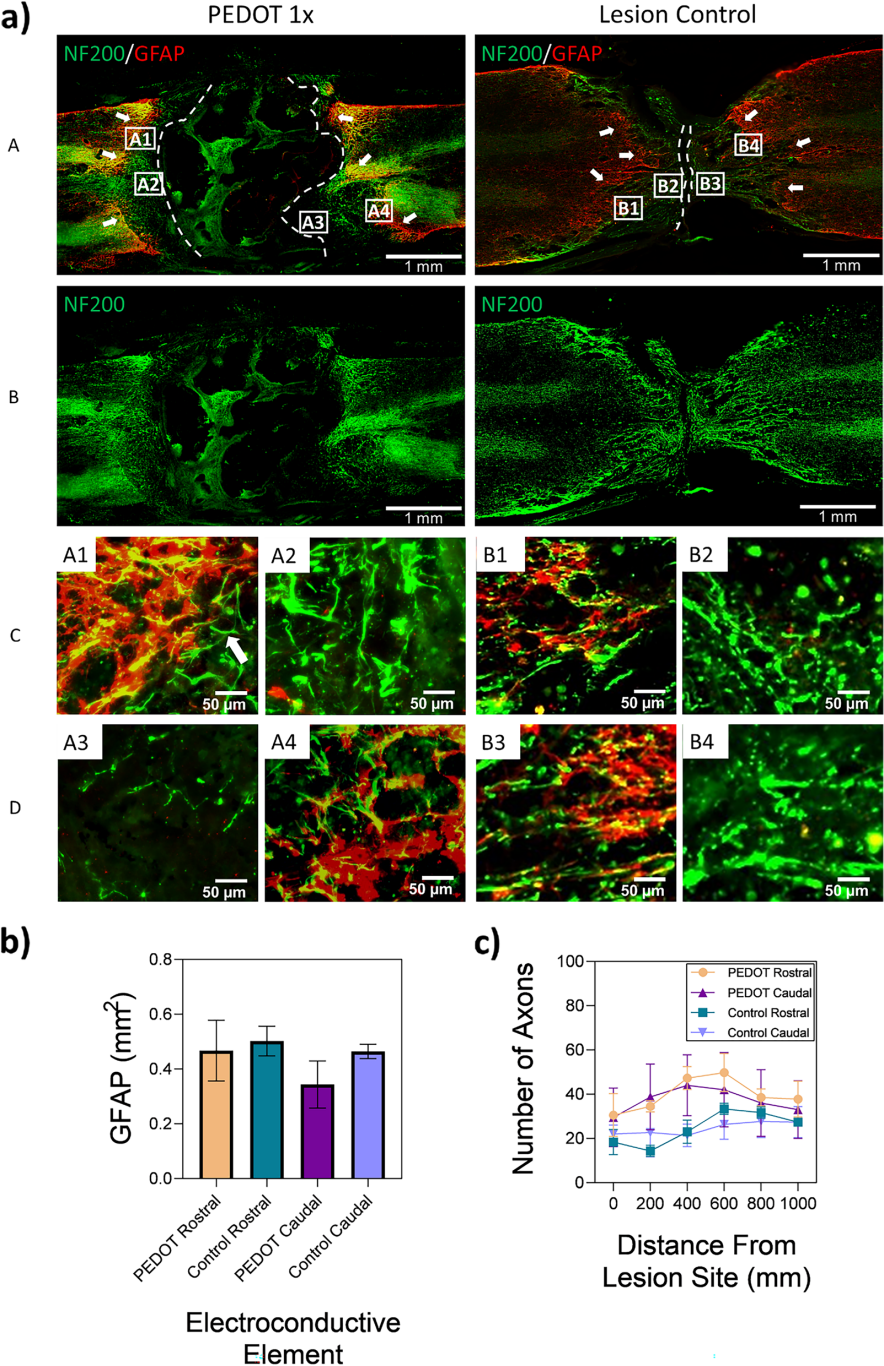
Gel:HA:PEDOT-NPs 1 × scaffold provided a diminished astrocyte reactivity around the lesion border and increased axonal migration towards the lesion site. **a** Representative immunofluorescence images of spinal cord tissue section labelling with NF200 (green) and GFAP (red) in rats implanted with PEDOT 1 × gel:HA:PEDOT-NPs and injured as lesion control (A-B), scale bar -1 mm. Dashed lines indicate the lesion/scaffold area and arrows indicate the periphery of GFAP reactive area. (C-D) Higher magnification of the area in rectangles in (A), scale bar -50 µm. **b** Quantification of the GFAP reactive area around the lesion site. c) Quantification of the number of axons from the lesion site in 200 µm distance intervals. * *p* < 0.05, (n ≥ 3, mean ± SD) is indicated where statistical difference is observed

Figure 10 (a-A) shows the spinal cord tissue stained by NF200 and GFAP markers. GFAP labelling of the spinal cord sections show an upregulation of astrocyte activity around the periphery of the lesion/implementation site (indicated by the dashed lines), as shown by arrows in Fig. 10 (a-A). The area of GFAP activation in the PEDOT 1 × scaffold is lower at 0.47 ± 0.1 mm^2^ at the rostral side when compared to the lesion only control at 0.5 ± 0.05 mm^2^ as shown in Fig. 10 (b), though no statistical difference between the groups was found. Furthermore, NF200 labelled axons can be observed passing through the GFAP reactivity boundary towards the scaffolds site at both rostral and caudal sites of the spinal cord, as shown in Fig. 10 (a-A,B), with an example of an axon indicated by an arrow in Fig. 10 (a-C, *A1)*. The number of axons present at 200 µm intervals from the lesion site are presented in Fig. 10 (c). A higher number of axons can be observed closer to the lesion site at both the rostral and caudal sides in the PEDOT 1 × scaffold when compared to the lesion only control. At 200 µm from the lesion site, 34.5 ± 2.65 axons were present on the caudal side of the PEDOT scaffold as compared to 14.3 ± 2.52 axons in the lesion only control, though no statistical difference between the groups was found.

**Fig. 11:**
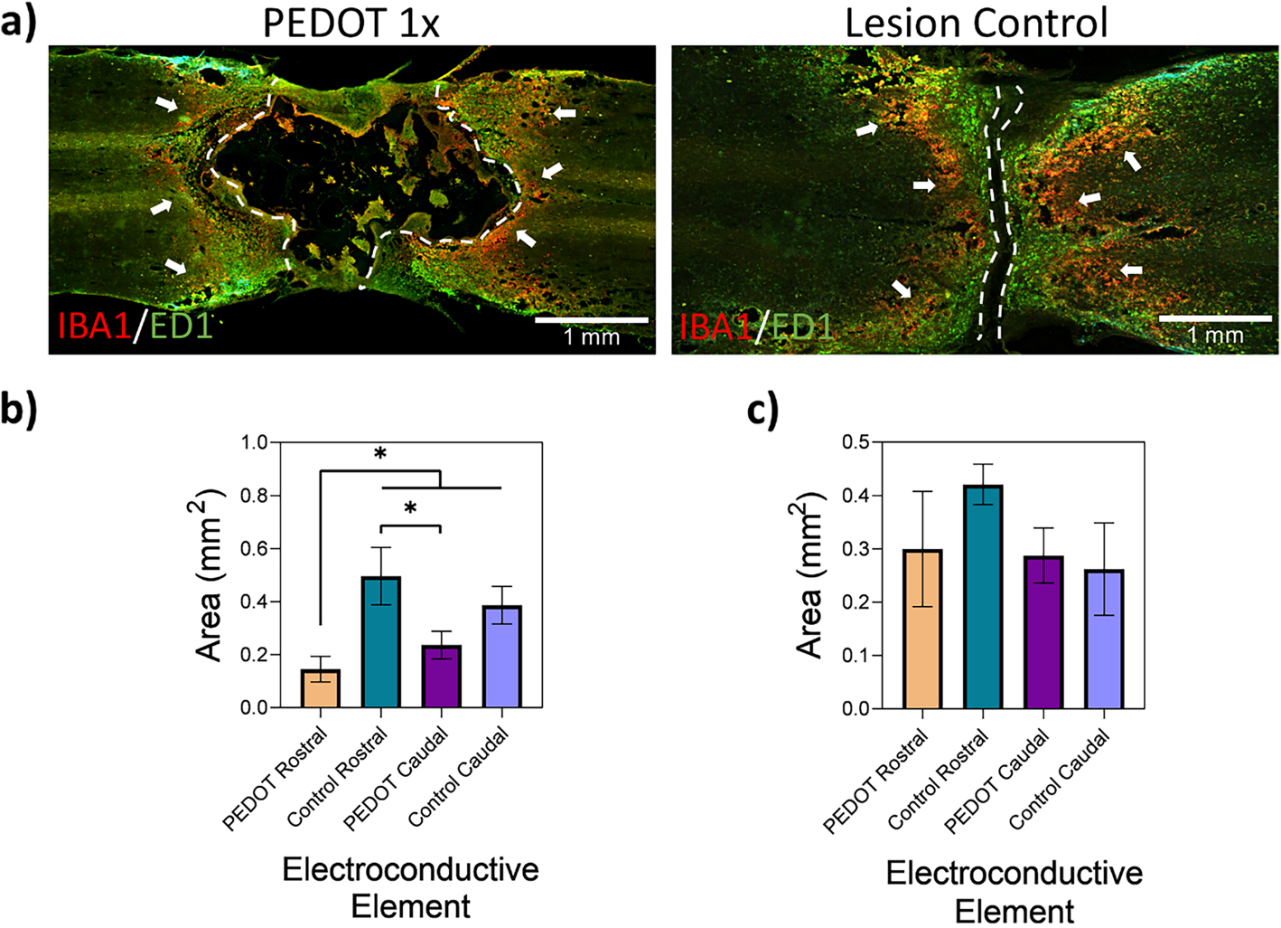
Gel:HA:PEDOT-NPs 1 × scaffold induced diminished macrophage and microglia reactivity and provided a more controlled inflammatory response and reactive area around the lesion site when compared to the lesion only control. **a** Representative immunofluorescence image of IBA1 (red) microglia and ED1 (green) macrophages reactivity observed in the PEDOT 1 × scaffold 4 weeks post injury on both rostral and caudal sides and the lesion only control, scale bar -1 mm. Dashed lines indicate the lesion/scaffold area and arrows indicate the periphery of IBA1/ED1 reactive area. **b** Quantification of the IBA1 reactive area from the lesion site. c) Quantification of the ED1 reactive area from the lesion site

Microglia and macrophage reactivity of the spinal cord tissues of interest can be observed in Fig. 11 (a), labelled by means of IBA1 (red) and ED1 (green), respectively, with the scaffold/lesion borders outlined by dashed lines. The microglia are not as heavily populated in areas directly in contact with the scaffold in the PEDOT 1 × group, particularly on the rostral side, as indicated by arrows. The area of microglia activation in the PEDOT 1 × scaffold is lower at 0.15 ± 0.05 mm^2^ at the rostral side when compared to the lesion only control at 0.5 ± 0.11 mm^2^ as shown in Fig. 11 (b), though no statistical difference between the groups was found. The presence of macrophages can in turn be directly observed closer to the periphery of the scaffold implant site. Lower inflammatory response can also be observed in the area of reactive macrophages present in the PEDOT 1 × scaffold, as is shown in Fig. 11 (c).

**Fig. 12:**
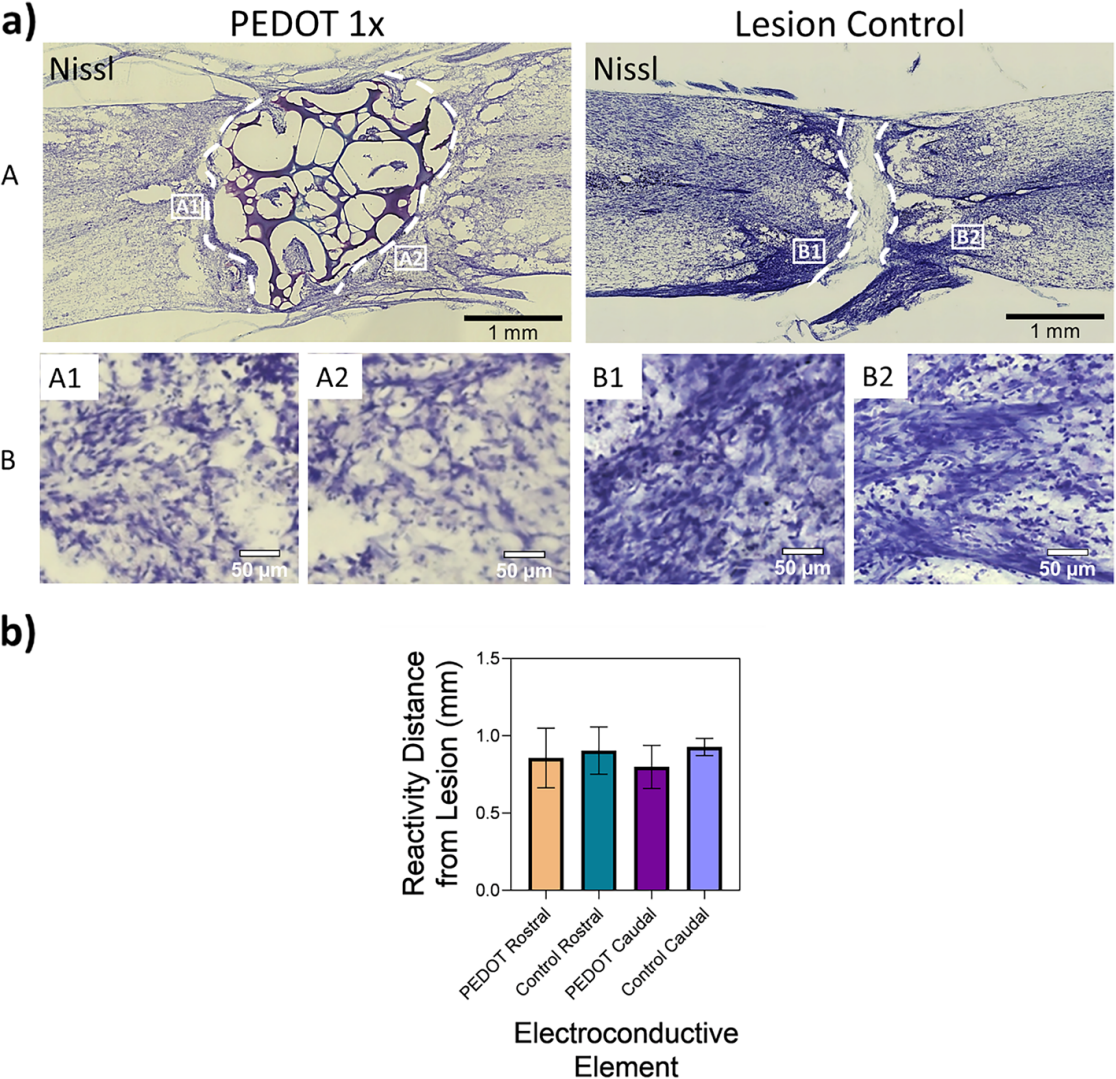
Gel:HA:PEDOT-NPs 1 × scaffold induced a more controlled inflammatory response and reactive area around the lesion site when compared to the lesion only control. **a** Representative images of spinal cord tissue section Nissl labelling in rats implanted with PEDOT 1 × gel:HA:PEDOT-NPs or injured as control (A), scale bar -1 mm. Dashed lines indicate the lesion/scaffold area. (B) Higher magnification of the indicated areas in (A), scale bar -50 µm. **b** Quantification of the distance of the Nissl reactive area from the lesion site. * *p* < 0.05, (*n* ≥ 3, mean ± SD) is indicated where statistical difference is observed

A similar observation can be made in the Nissl staining of the tissues, as shown in Fig. 12 (a), with the scaffold/lesion borders outlined by dashed lines. Higher reactivity is present in the lesion control at both sides of the injury when compared to the PEDOT 1 × scaffold group. For example, the reactivity distance from the lesion site is greater in the lesion control on caudal side at 1.02 ± 0.2 mm than in the PEDOT 1 × scaffold at 0.8 ± 0.14 mm, as shown in Fig. 12 (b), though no statistical difference between the groups was found.

## Discussion

The aim of this study was to characterise the developed gel:HA:PEDOT-NPs scaffolds and also investigate their initial biocompatibility in-vitro and in-vivo in rat SCI models. Gelatin and HA biomaterials were successfully incorporated with novel conductive PEDOT NPs to create gel:HA:PEDOT-NPs. Utilisation of the PEDOT NP solutions of different NP concentrations, as shown in Table 1, to create gel:HA:PEDOT-NPs hydrogels resulted in very viscous solutions. The addition of HA significantly increased the viscosity due to its high molecular weight. To obtain a smooth solution for mould casting, air bubbles trapped in the solution during the mixing stage were removed from the hydrogels by means of ultrasonication and centrifugation.

### Morphological characterisation of PEDOT scaffolds

Morphological analysis showed that the PEDOT NPs were distributed evenly throughout the scaffolds and the scaffolds possessed an overall porous internal structure (Fig. 2 (a)). Typically, conducting polymers show limited biodegradability due to the fact that they possess poor solubility. Strategies to improve the biodegradability include graft co-polymerisation and blending with biodegradable polymers such as PLA, chitosan and PLGA [41, 42]. In the present study PEDOT is nanostructured and waterborne, therefore the biodegradability is enhanced. The slow freezing step in the manufacturing process allows for the formation of large ice crystals which results in large pores post lyophilisation. The difference in the porosity between PEDOT 1 × NP scaffold and lower PEDOT concentrations could be attributed to the hydrophobic nature of the NPs, leading to differences in ice morphology, thus creating smaller pores [43, 44]. As one of the main reasons for inclusion of pores in TE scaffolds includes nutrient and oxygen diffusion to support cellular viability, porosity must be carefully controlled to match the cellular constraints. Pores ranging around 200–300 µm have been shown to support proliferation rates of MSCs in-vitro, with the porosity of the gel:HA:PEDOT-NPs scaffolds residing within these parameters [45–47], as shown in Fig. 2 (b).

### Mechanical characterisation of PEDOT scaffolds

To create a biomimetic scaffold, the mechanical properties of the scaffold should closely match that of the native tissue. The mechanical properties of the gel:HA:PEDOT-NPs scaffolds have been carefully tailored by modulating the gelatin concentration and/or crosslinking efficiency to match the Young’s Modulus of the native spinal cord which is 0.8–1.37 MPa [6, 7, 11], Fig. 3 (a).

### Swelling degree of PEDOT scaffolds

Swelling studies of the gel:HA:PEDOT-NPs scaffolds were conducted to determine the EDC/NHS crosslinking efficiency and are shown in Fig. 3 (b). Crosslinking of gelatin based scaffolds by means of EDC and NHS modification is a non-zero chemical crosslinking regime which activates carboxyl groups and forms an intermediary, which reacts with primary amino acids on adjacent gelatin chains, a process which is then stabilised by the addition of NHS to the crosslinking regime [35]. The increase in the swelling degree of the PEDOT NPs samples over the 96 h period could potentially be explained by a decrease in the crosslinking efficiency due to the presence of the NPs. Conversely, the swelling profile of the control samples began to decrease slightly after 24 h indicating the onset of hydrolytic degradation.

### Conductive characterisation of PEDOT scaffolds

As the main aim of developing gel:HA:PEDOT-NPs scaffolds was to create electroconductive scaffolds for SCI repair, the scaffolds were analysed via dielectric measurements and shown in Fig. 4 (a-b). The change in the electrical conductivity with varying PEDOT NP concentrations can be explained due to the differences in the Gel:HA matrix, which is not as efficiently crosslinked, allowing better contact between PEDOT NPs compared to the samples with lower concentrations. Hydrated samples exhibit higher conductivity values due to ionic contributions. For example, samples with 0.25 × and 0.5 × NPs display similar conductivity values (5.8 × 10^–4^ and 4.3 × 10^–4^ S/cm, respectively) with 1 × PEDOT samples again exhibiting increased conductivity, indicating easier ion mobility as result of increased concentrations of PEDOT NPs. The hydrated samples in this instance should be regarded with the most consideration as the goal of most TE scaffolds is to be implanted *in-situ*, with hydration of the scaffolds occurring by means of the surrounding body fluids. Tissues are considered volume conductors by means of ion flux, thus the combination of ionic and electrically conductive influences in the hydrated PEDOT scaffolds from both the PEDOT NPs and high water concentration could aid to bridge the gap between electrical and cellular conductivity interactions [48, 49]. Conductivity values in the PEDOT samples are also higher for the crosslinked samples than for the non-crosslinked samples. Permittivity decreases as the PEDOT NP concentration increases, for both non-crosslinked and crosslinked samples. However, for the re-hydrated samples the trend is the opposite. It may be speculated that the crosslinking regime facilitates the conduction mechanism. These results reveal that the morphological changes after crosslinking are important in controlling both conductivity and permittivity of the samples, and how the presence of the hydration medium translates into an increase in both the conductivity and the dielectric permittivity.

### Rheological analysis of PEDOT hydrogel

3D printing for SCI repair is an alternative route to the mould casting manufacturing method to create scaffolds of various architecture [50]. The two main characteristics of a printable material are their shear-thinning ability, as well as shape fidelity post-printing. Rheological analysis (Fig. 6 (c)) showed that the gel:HA:PEDOT-NPs hydrogels possessed a shear thinning behaviour. To also investigate the latter, recovery profiles of the gel:HA:PEDOT-NPs hydrogel samples were gathered (Fig. 6 (d)), whereby the hydrogels were subjected first to very low shear rates of 0.1 s^−1^, which simulates the sample in the printing cartridge. Then the shear was rapidly increased to 100 s^−1^ for 10 s which mimics the high shear forces exhibited by the material while extruded from the nozzle during the printing process. The shear rate was then rapidly decreased back to 0.1 s^−1^ to replicate the removal of the shear force once the material is printed. The time taken to increase the viscosity post-printing informs on the potential of the material to have high shape fidelity post-printing, which will allow for the printed geometry to be retained before the crosslinking regime can be introduced. For instance, the initial viscosity of the PEDOT 1 × sample was 3383.6 Pa.s, which decreased to 15.9 Pa.s with the application of the higher shear rate. 5 s after the high shear was removed, the viscosity increased again to 495.6 Pa.s, with a 14.6% recovery rate of the initial viscosity. 10 s and 20 s time recovery allowed the sample to recover 21.3% and 69.4%, respectively, suggesting that the majority of the recovery window occurs during the initial 20 s post-printing due to the reorganization of the polymer chain network alignments [15, 31]. Overall, the addition of the NPs into the Gel:HA hydrogel increased the ability of the material to recover when compared to the control. Examples of 3D printed gel:HA:PEDOT-NPs can be found in Figure S2 in the Supplementary Information.

### Preliminary cytocompatibility assessment of gel:HA:PEDOT-NPs scaffolds in-vitro

Gel:HA:PEDOT-NPs scaffolds were initially examined for their biocompatibility in-vitro with MSCs and the Alamar Blue***™*** proliferation assay. Overall, the constant proliferation rates of the MSCs over the 96 h period suggests that the gel:HA:PEDOT-NPs scaffolds are not cytotoxic. Lower proliferation rates of MSCs between the scaffolds samples and the cell control can be potentially explained by experimental set-up, whereby a small percentage of the seeded cells were washed away during the seeding process, even with great care taken to minimise this. The smaller cell numbers correlate with a smaller Alamar Blue***™*** Reduction in the scaffold groups.

To further visualise and quantify the attachment and proliferation of MSCs seeded on gel:HA:PEDOT-NPs scaffolds, LIVE/DEAD staining was conducted. Overall, high viability of cells was observed across all groups. The arrangement of DiI/DAPI stained MSCs on the scaffolds is dictated by the surface topography of the scaffolds, which is not smooth, as shown in Fig. 2 (a-A). MSCs tend to adhere to the scaffolds in clusters within the surface pores and grooves, thus creating a network of cellular groups rather than allowing for a homogenous cell spread throughout the surface of the scaffold to occur. The cluster distribution of the MSCs within the surface topography of the scaffolds is reiterated in both the LIVE/DEAD staining and DiI/DAPI staining, showing the typical MSCs spindle like morphology (Fig. 7 (c-A,B) and Fig. 8 (a-A, B and D), respectively). Neuronal Stem Cells (NSCs) stained with tdTomato were also seeded on the gel:HA:PEDOT-NPs scaffolds, fixed and stained with DAPI, as shown in Figure S3. NSCs proliferation and growth on the scaffolds was similar to that of MSCs. The various in-vitro experiments further indicate that the gel:HA:PEDOT-NPs scaffolds of varying PEDOT NP concentrations are biocompatible and support the survival and proliferation of MSCs in-vitro. This is mostly contributed to the high gelatin concentration in the formulation of the scaffolds, which aids in the attachment of cells onto the surface of the scaffold by means of cell adhesive ligands and motifs such as RGD and GFOGER peptide sequences present in the gelatin structure [15, 51–53].

### Preliminary cytocompatibility assessment of gel:HA:PEDOT-NPs scaffolds in-vivo

To further study the in-vivo biological response of the gel:HA:PEDOT-NPs scaffolds, the PEDOT 1 × scaffold was chosen to be implanted into a full transection rat SCI model at the T3 level (Fig. 9) for a period of four weeks. As this phase of the experimental set-up was established as an initial screening of the effectiveness and biocompatibility of the incorporation of PEDOT NPs into biomaterials in-vivo, small sample sizes of animals and scaffold formulations were used, with the lesion only model acting as the control group, which are the limitations of the current study. The PEDOT 1 × scaffold formulation was chosen for the initial in-vivo biocompatibility study due to its most favourable viability response when tested in-vitro with MSCs.

GFAP is a sensitive marker which detects astrocytes that are responding to insults of the CNS. GFAP activation in SCI lesions is usually aligned closely with the site of injury. It is proposed that the phenotypic transformation from reactive astrocytes into scar forming astrocytes is influenced by signals from the lesion site, evidenced by the decreasing density gradient of scar forming astrocytes away from the lesion site [4, 54, 55]. Hence, the unusual placement of the GFAP upregulation in Fig. 10 (a-A) which is not directly in contact with the lesion/implantation site is interesting to note, as well as its diminished intensity in the astrocyte reactivity when compared to the lesion control at both the rostral and caudal sides of the lesion.

The pattern of the IBA1 microglia labelling shown in Fig. 11 (a) follows a similar pattern to that of the GFAP labelling observed in Fig. 10 (a-A). Microglia are one of the most sensitively activated responders during injuries to the CNS and play a major role in the clearing of debris and phagocytosis during the systemic inflammatory response [4, 56]. The activation of the microglia, which can last up to 180 days following the initial SCI, along with the subsequent release of cytokines, play a major role in the activation of astrocytes and thus GFAP expression [4, 57]. The role of debris clearance of the monocyte-derived macrophages is similar to that of microglia and is essential to recovery. Also just like the microglia, the presence of pro-inflammatory M1 macrophages also influences the activation of reactive astrocytes [4, 56]. Overall, the presence of inflammatory cells is lower in the PEDOT 1 × scaffold than in the lesion only control, indicating that the implanted scaffold is having a positive influence in terms of re-establishing signals and initiation of healing mechanisms.

It is known that the presence of inflammatory responders such as reactive astrocytes promote the formation of the glial scar which does not aid recovery. However, numerous studies have also pointed to the benefits of glial scar formation as a controller of inflammation [4, 5], and further that the reduction or elimination of scar formation does not promote axon regrowth in severe SCI and leads to worse functional recovery [58]. Hence, the presence of the GFAP reactive periphery in combination with microglia and macrophages reported here should be examined using this dual lens. While reactivity around the lesion site of the PEDOT 1 × scaffold does exist, it appears to be much more controlled in its inflammatory response when compared to the lesion control. The presence of inflammatory cells is common in any injury, particularly one as traumatic to the system as a full transection of the spinal cord, though in the case of SCI the presence of uncontrolled inflammatory responses is detrimental as it causes further neuronal loss [4].

It could be hypothesised that a controlled inflammatory response, which includes reduced microglia and macrophage activity, is influenced by the presence of HA component in the scaffold material. HMW-HA has been shown to limit astrocyte activation through CD44 receptors and thus also limit scar formation [11, 59]. HMW-HA also lowers the numbers of immune cells such as microglia and macrophages in SCI rat lesion models [11, 60]. The combination of HA and electroconductive PEDOT NPs in the scaffold group could have allowed for a relatively greater axonal migration and growth towards the targeted implantation site by providing a more stimulating environment for regeneration.

Even though no axons can be observed passing into the PEDOT scaffolds, they appear to be drawn towards the scaffold. The scaffold contains internal pores which are not accessible from the outside surface, which is a limitation of the current gel:HA:PEDOT-NPs scaffolds architecture. This is believed to diminish the possibility of axons to grown through the lesion site without channels in the scaffold to guide the re-growth of cells effectively. Improved axonal re-growth and functional recovery have been reported in SCI regeneration studies with conductive scaffolds, though the exact mechanism which prompts this regeneration is not fully elucidated to date [61, 62]. Although it was impossible to obtain high magnification images, there is no evidence of cellular uptake of NPs within the time period of this study.

The previously reported results correlate well with the in-vivo study presented here [61, 63], such as the study conducted by Shu et al*.* whereby Polylactic acid (PLA) and polypyrrole scaffolds were implanted into rat lesion SCI models. The results also similarly indicated the decrease in the GFAP positive astrocytes around the lesion areas, as well as an increase in NF200 stained axons six weeks post-implementation in the presence of the conductive scaffold [61].

## Conclusions

The tailored synthesis of the PEDOT NPs without toxic PSS has allowed for the development of NPs which could be homogeneously distributed within the struts of a hydrogel based tissue scaffold without biocompatibility issues. The scaffold itself is based on a blend of gelatin and HA which has been designed for optimal porosity, with biomimetic mechanical performance and with high conductivity to simulate axons for regeneration purposes. The NPs and scaffolds induced moderate inflammatory reaction in-vivo which was less severe than the observed response in animals with lesion only, suggesting that the toxic environment was attenuated. These promising results, including GFAP upregulation not being directly at the lesion/implantation site and diminished astrocyte reactivity, are attributed to the scaffold influence on the initiation of the regeneration processes via decreased levels of macrophages and microglia at the implant site. Axonal attraction towards the scaffold can be attributed to a controlled inflammatory response which is immunomodulated by the limitation of astrocyte activation through CD44 receptors of HA, which limits scar formation at the implant site. Overall, the structure/property/function relationships of these scaffolds have been carefully tailored to optimise their performance at the site of injury and they show enormous promise in promoting repair and regeneration for future applications.

## Supplementary Information

**Additional file 1. **

## Data Availability

The datasets used and/or analysed during the current study are available from the corresponding author on reasonable request.

## Abbreviations

CNS: Central Nervous System
ECM: Extracellular Matrix
EDC: N-(3-Dimethylaminopropyl)-N′-Ethylcarbodiimide Hydrochloride
EDOT: Ethylenedioxythiophene
Fe-Tos: Iron(III) P-Toluenesulfonate Hexahydrate
Gel:HA: Gelatin And Hyaluronic Acid
GFAP: Glial Fibrillary Acidic Protein
HA: Hyaluronic Acid
HMW-HA: High Molecular Weight Hyaluronic Acid
MSCs: Mesenchymal Stem Cells
MEMα: Minimum Essential Medium Alpha
NHS: N-Hydroxysuccinimide
NP: Nanoparticles
PANI: Polyaniline
PBS: Phosphate Buffered Saline
PDADMAC: Poly(Diallyldimethylammonium Chloride
PFA: Paraformaldehyde
PEDOT: Poly(3,4-Ethylenedioxythiophene)
PEDOT:PSS: Poly(3,4-Ethylenedioxythiophene) Polystyrene Sulfonate
PEDOT NP: Poly(3,4-Ethylenedioxythiophene) Nanoparticles
PLA: Polylactic Acid
PPy: Polypyrrole
SCI: Spinal Cord Injury
TE: Tissue Engineering
