# Supplementary material for: Electroconductive PEDOT nanoparticle integrated scaffolds for spinal cord tissue repair
Source: Biomater Res. 2022 Nov 22;26:63. doi: 10.1186/s40824-022-00310-5 (PMC9682832; doi:10.1186/s40824-022-00310-5)
Supplement: Supplementary file 1 — Additional file 1. [file 40824_2022_310_MOESM1_ESM.docx]

**Supporting Information**

**Electroconductive PEDOT Nanoparticle Integrated Scaffolds for Spinal Cord Tissue Repair**

**Aleksandra Serafin^1^, Mario Culebras Rubio^2^, Marta Carsi^3^, Pilar Ortiz-Serna^4^, Maria J. Sanchis^4^, Atul K. Garg^5^, J. Miguel Oliveira^6,7^ Jacob Koffler^8,9^, Maurice N. Collins^1,10^**

^1^School of Engineering, Bernal Institute, University of Limerick, Limerick, Ireland;

^2^Materials Science Institute (ICMUV), Universitat de València, c/ Catedrático José Beltrán 2, 46980 Paterna, Valencia, Spain;

^3^Instituto de Automática e Informática Industrial, Universitat Politècnica de Valencia, 46022 Valencia, Spain;

^4^Instituto Tecnológico de la Energía, Departamento de Termodinámica Aplicada, Universitat Politècnica de València, Camí de Vera s/n, 46022, Valencia, Spain;

^5^Manufacturing Technology and Innovation Global Supply Chain, Johnson & Johnson, Bridgewater, New Jersey 08807, USA

^6^3B’s Research Group, I3Bs—Research Institute on Biomaterials, Biodegradables and Biomimetics, University of Minho, Headquarters of the European Institute of Excellence on Tissue Engineering and Regenerative Medicine, AvePark, Parque de Ciência e Tecnologia, Zona Industrial da Gandra, 4805-017 Barco, Guimarães, Portugal;

^7^ICVS/3B’s—PT Government Associate Laboratory, 4710-057 Braga, Braga, Portugal; ^8^Department of Neuroscience, University of California San Diego, La Jolla, CA, USA 92093;

^9^Veterans Affairs Medical Center, San Diego, CA, USA;

^10^Health Research Institute and AMBER, University of Limerick, Limerick, Ireland.

***
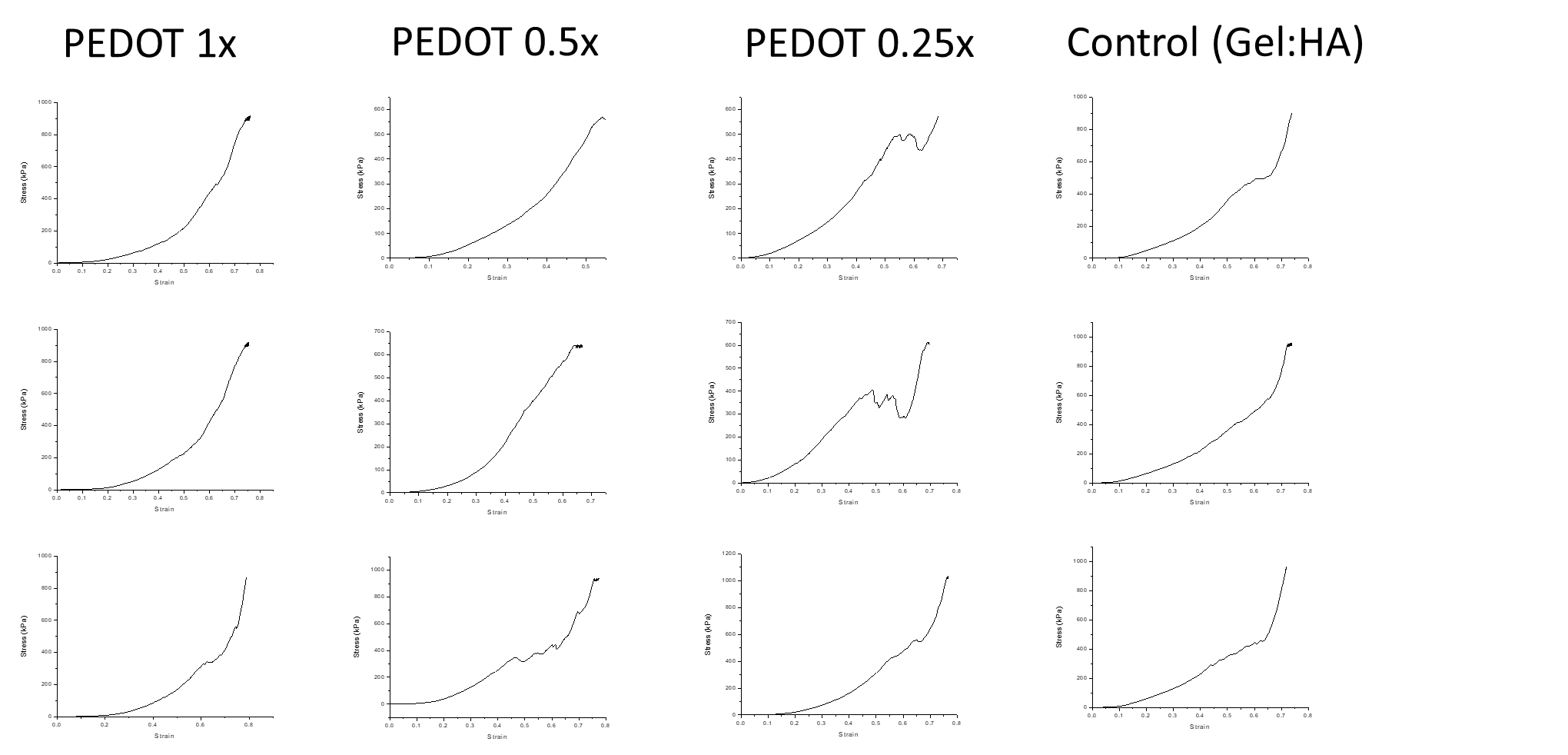
***

***Figure S1: Stress vs. Strain graphs of gel:HA:PEDOT-NPs scaffolds from which the Young’s Modulus was derived by taking the slope in the linear region.***


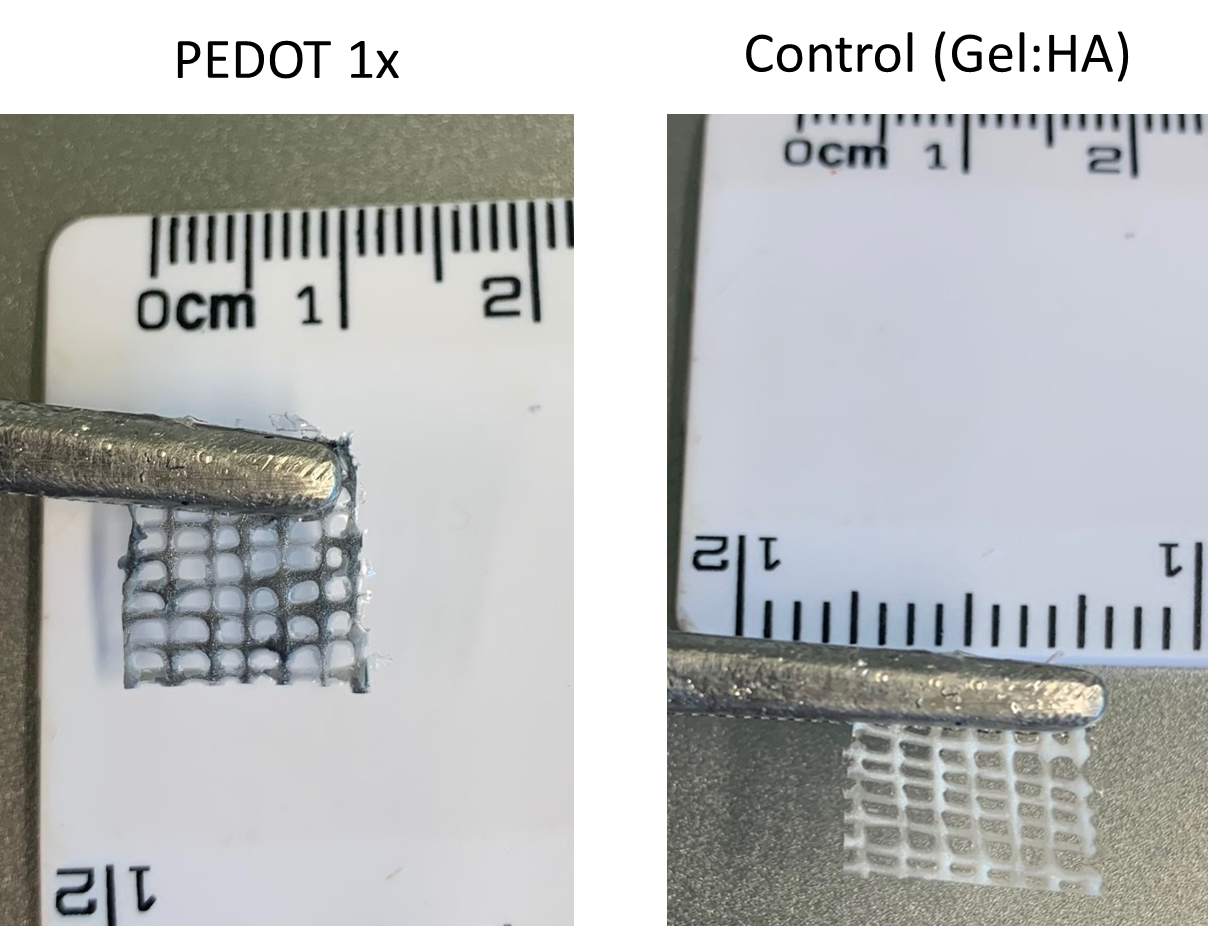


***Figure S2: Representative 3D printed lattice of the PEDOT 1× and Gel:HA (control with no PEDOT NPs) hydrogels before a crosslinking regime is introduced.***

***In-Vitro Neuronal Stem Cell Assessment***

tdTomato Neuronal stem cells (NSCs) were grown in ENStem-A™ Neural Expansion Medium, supplemented with L-Glutamine (2 mM), FGF-2 (2 ng/mL) and 1% Penicillin-Streptomycin in a 5% CO_2_ environment.

tdTomato NSCs were also used to study the morphology and attachment onto Gel:HA:PEDOT-NP scaffolds. Prior to NSCs seeding, Gel:HA:PEDOT-NP scaffolds were coated with 20 µg/mL Poly-D-Lysine (PDL) and 10 µg/mL laminin to allow for cellular attachment. NSCs were seeded at a density of 0.25x106 cells per scaffold onto pre-conditioned Gel:HA:PEDOT-NP scaffolds in a 24-well plate, with the media changed every 48 h. After a period of 96 h, the cells were fixed with 4% PFA, stained with DAPI (1:1000), imaged and shown in Figure S3.

The gel:HA:PEDOT-NP scaffold are intended to act as a bridge for regeneration in-vivo as the amount of NSCs in the spine is low, therefore the assessment of their differentiation within the scaffold was not conducted.


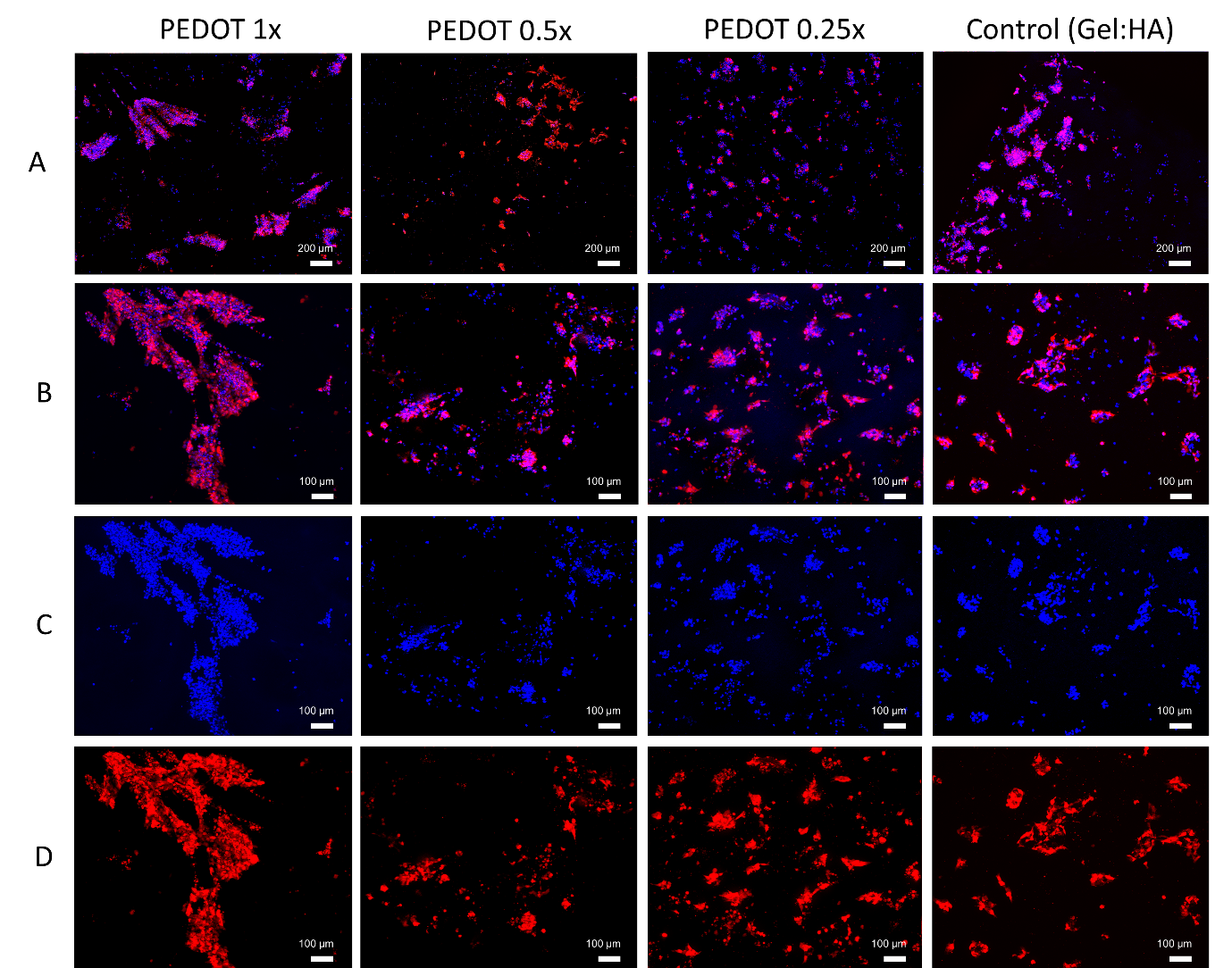


***Figure S3: NSCs stained with tdTomato (red) seeded onto gel:HA:PEDOT-NPs scaffolds of different NP concentrations and cultured for a period of 96 h, fixed and stained with DAPI (blue), (A) scale bar - 200 µm. (B,C,D) scale bar - 100 µm.***
